# Uncovering the signatures of aging and senescence in the human dorsolateral prefrontal cortex

**DOI:** 10.1016/j.xgen.2025.101127

**Published:** 2026-01-22

**Authors:** Nicholas X. Sloan, Jason Mares, Aidan C. Daly, Shaunice Grier, Imdadul Haq, Christopher A. Jackson, Natalie Barretto, Obadele Casel, Kristy Kang, Shruti Khiste, Kennedy Harris, Jacqueline Eschbach, Benjamin T. Fullerton, Courteney Mattison, Brhan Gebremedhin, Joana Petrescu, Lilian Coie, Maria Hauge Pedersen, Ke Zhang, Jian Shu, Andrew F. Teich, Hasini Reddy, Colin P. Smith, Yousin Suh, Vilas Menon, Hemali Phatnani

**Affiliations:** 1 Department of Neurology, Columbia Irving Medical Center, New York, NY, USA; 2 Motor Neuron Center, Columbia University, New York, NY, USA; 3 Center for Genomics of Neurodegenerative Disease, New York Genome Center, New York, NY, USA; 4 Center for Translational and Computational Neuroimmunology, Columbia Irving Medical Center, New York, NY, USA; 5 Cutaneous Biology Research Center, Massachusetts General Hospital, Harvard Medical School, Boston, MA, USA; 6 Broad Institute of MIT and Harvard, Boston, MA, USA; 7 Harvard-MIT Program in Health Sciences and Technology, MIT, Cambridge, MA, USA; 8 Department of Pathology and Cell Biology, Columbia University, New York, NY, USA; 9 Taub Institute for Research on Alzheimer’s Disease and the Aging Brain, Columbia University, New York, NY, USA; 10 Centre for Clinical Brain Sciences, The University of Edinburgh, EH16 4SB Edinburgh, UK; 11 Department of Obstetrics and Gynecology, Columbia University, New York, NY, USA; 12 Department of Genetics and Development, Columbia University, New York, NY, USA

**Keywords:** aging, senescence, prefrontal cortex, transcriptome, spatial transcriptomics, single-nucleus transcriptomics

## Abstract

We performed Visium spatial transcriptomics (ST) and single-nucleus RNA sequencing (snRNA-seq) on a cohort of nonpathological human tissues to uncover signatures of aging and senescence in the dorsolateral prefrontal cortex (dlPFC). In doing so, we identified gene expression changes characteristic of aged cortical layers. The cellular composition of the dlPFC also changed with age, with increased homeostatic astrocyte abundance and with decreased somatostatin (*SST*) inhibitory neurons. Nuclei from dlPFC cell types displayed a strong decline in oxidative phosphorylation- and cytoplasmic translation-related genes with age. Additionally, oligodendrocytes showed several hallmarks of senescence and a linear increase in *CDKN2A* expression with age. Combined analysis of ST and snRNA-seq datasets revealed astrocyte- and vascular cell-related gene expression programs in the white matter and layer 1 that were strongly enriched with age and for senescence-associated genes. These findings will help facilitate future studies exploring the role of senescent cell subpopulations in the aging brain.

## Highlights

• In the aging dlPFC, we observe a loss of *SST* transcript and *SST*+ neuron density
• Loss of nuclear oxidative phosphorylation- and cytoplasmic translation-related genes
• Increased senescence hallmarks and *CDKN2A* transcript in oligodendrocytes
• An enrichment of spatially coordinated astrocyte and vascular cell gene programs

## Graphical abstract

**Figure:**
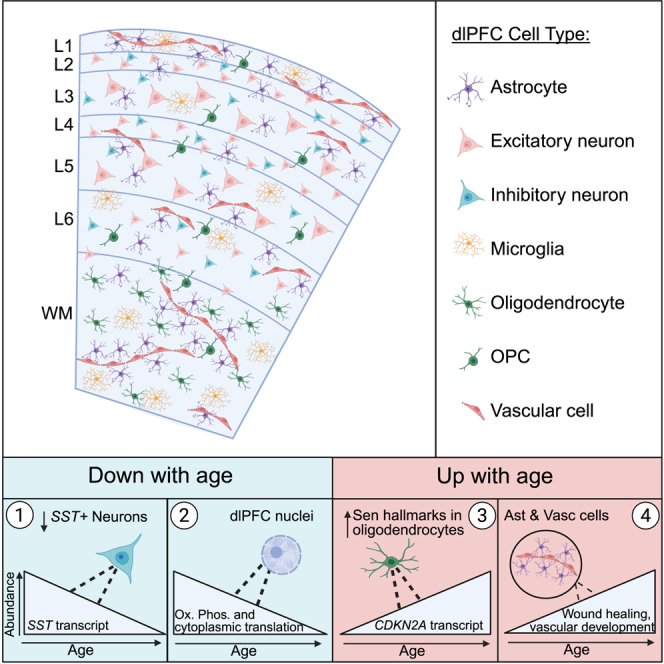

## Teaser

A transcriptomic atlas of aging and senescence in the brain across the adult human lifespan is built utilizing 55 nonpathological dlPFC samples profiled using spatial transcriptomics and single-nucleus RNA sequencing. This atlas reveals important insights into how cortical layers and the cell types residing within them change with age.

## Introduction

The dorsolateral prefrontal cortex (dlPFC) is a brain region that regulates executive functions such as working memory, planning, and maintaining attention.^1^^,^^2^ Adults display worse performance in tasks that assess these key executive functions throughout aging.^3^^,^^4^^,^^5^^,^^6^^,^^7^ Additionally, this loss of cognitive function is accompanied by a decline in cortical integrity, as PFC volume decreases at an average rate of 5% every 10 years after age 40.^8^ While such gross structural changes of the cortex are well established, finer differences in cellular composition and gene expression programs with age are less clear. The laminar organization of the dlPFC is known to be intimately tied to its function,^9^^,^^10^ with six cortical layers defined by unique combinations of cell types, which in turn display distinct patterns of gene expression and connectivity. With the rise of new spatial and single-cell transcriptomic technologies, we are now uniquely positioned to investigate how the layers of the dlPFC and the cell types that reside within them change with age.^11^^,^^12^ Such technologies also permit the investigation of rare cell-type subpopulations driving the aging process, such as senescent cells.

Cellular senescence, a central hallmark of aging,^13^^,^^14^ encompasses heterogeneous cellular states triggered by prolonged cell stress resulting in the irreversible arrest of proliferation and permanent changes to cell function *in vivo*.^15^^,^^16^^,^^17^^,^^18^^,^^19^ Two main tumor-suppressive pathways establish and maintain senescence: one governed by p53 and p21 (*CDKN1A*), the other by pRB and p16 (*CDKN2A*).^20^ Both pathways converge on downstream inhibition of CDK4 and CDK6 kinases, preventing cell-cycle progression from G1 to S phase. Senescent cells contribute to aging and age-related pathologies^21^^,^^22^^,^^23^ through their senescence-associated secretory phenotype (SASP).^24^^,^^25^^,^^26^ The SASP is composed of pro-inflammatory cytokines, chemokines, exosomes, growth factors, and extracellular matrix-degrading proteins that are released into the surrounding tissue environment following the induction of growth arrest.^24^^,^^25^^,^^26^^,^^27^^,^^28^^,^^29^ Much of the diverse roles senescent cells play throughout the body can be attributed to differences in SASP composition, as separate senescent cell subpopulations have been theorized to promote tissue regeneration and inflammation.^24^^,^^30^ The SASP has also been shown to have paracrine activities, inducing senescence in nearby cells.^24^

In addition to being rare and heterogeneous in presentation, senescent cells accumulate in aged tissues,^31^^,^^32^^,^^33^ including the brain.^34^^,^^35^^,^^36^ Further, whole-body clearance of senescent cells in mice, either pharmacologically using dasatinib and quercetin (D + Q) or genetically in INK-ATTAC (p16INK4a apoptosis through targeted activation of caspase 8)^37^ mice treated with AP20187, resulted in improved cognitive function and extended healthy lifespan.^38^ However, despite their importance, much of the evidence supporting the existence of senescent cells within the healthy human brain has measured the abundance of one or a few senescence markers at a time. Indeed, full-scale transcriptomic or proteomic profiles of senescent cell populations in the brain have yet to be established. This is in part due to a scarcity of available study cohorts and an inability to collect *in situ* omics data with sufficient depth and throughput to locate the small percentage of cells that exhibit a senescent phenotype. Since no single marker is distinctive of senescence, the current recommendation of the NIH Common Fund’s Senescence Network (SenNet) is to identify senescent cells by the presence of multiple known hallmarks at once.^39^ In addition to cell-cycle arrest and the SASP, among these nine proposed hallmarks are the DNA damage response (DDR) pathway, nuclear changes, a resistance to apoptosis, increased lysosomal content, metabolic adaptations, cell surface markers, and changes in cell morphology.^39^

In this study, we applied spatial and single-nucleus genomics tools at scale across multiple tissues obtained from the University of Edinburgh Sudden Death Brain Bank and the New York Brain Bank at Columbia University to generate an atlas of aging and senescence in the human dlPFC. In doing so, we determined changes in single genes and cellular composition characteristic of aged cortical layers. Among these changes, we identified a loss of somatostatin (*SST*) transcript and *SST* inhibitory neuron abundance within aged gray-matter layers. We then characterized single-nucleus gene expression changes, which suggest a loss of genes related to oxidative phosphorylation and cytoplasmic translation shared across dlPFC cell types from old donors. Additionally, a higher propotion of oligodendrocyte nuclei were positive for senescence hallmarks like cell-cycle arrest and *CDKN2A* transcript with age. We then uncovered changes in spatiotemporally coordinated gene expression programs in the dlPFC. While spatial gene expression modules related to oxidative phosphorylation and deep-cortical-layer synaptic signaling decreased with age, wound healing and vascular-development modules increased with age and contained senescence-related genes. Finally, we determined how senescence hallmarks present within local neighborhoods of the dlPFC, and identified the white matter as a hotspot for senescent cell induction.

## Results

### Building a transcriptomic map of aging and senescence across the adult human lifespan

Postmortem dlPFC tissue (Brodmann areas 9 and 46) was collected from 55 donors with ages ranging from 27 to 89+ years (Table S1). This age-diverse cohort was divided into young (<45 years old, *n* = 12 donors), middle (45–55 years old, *n* = 20 donors), and old (>55 years old, *n* = 23 donors) age groupings. In-depth characterization of tissue samples and clinical data ensured that subjects were cognitively normal and lacked disease pathology.

**Figure 1:**
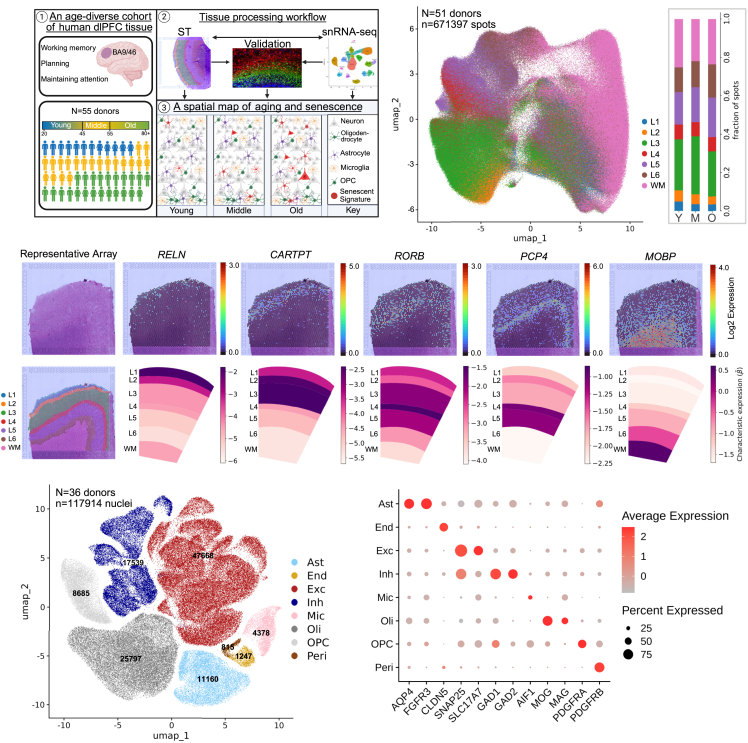
Building a transcriptomic map of aging and senescence in the dlPFC (A) Diagram outlining our project aims (generated in BioRender^42^). (B) UMAP embedding of ST data colored by AAR (left, L1–6, layer 1–6; WM, white matter). Bar plot depicting proportion of spots in each AAR for young (Y), middle (M), and old (O) donors (right). (C) H&E stain (top left) alongside manual annotation (bottom left) of a representative middle aged ST array. Spot-level gene expression of each layer marker measured in log-scaled counts on a representative array (top row, left to right). AAR-level Splotch-predicted expression (β)—measured in average log-scaled counts of gene expression per spot within an AAR of interest—of key cortical layer markers within the middle group (*n* = 19 donors; bottom row, left to right). (D) UMAP embedding of snRNA-seq data colored by broad class cell-type annotation. Number of nuclei profiles for each class displayed on top of each corresponding cluster in the UMAP (Table S3). Ast, astrocytes; End, endothelial cells; Exc, excitatory neurons; Inh, inhibitory neurons; Mic, microglia; Oli, oligodendrocytes; OPC, oligodendrocyte precursor cells; Peri, pericytes. (E) Average normalized expression level of key cell-type markers for annotated broad classes.

To build a transcriptomic map of aging and senescence (Figure 1A), we performed Visium spatial transcriptomics^40^^,^^41^ (ST) and single-nucleus RNA sequencing (snRNA-seq) on donor tissues throughout our assembled cohort. Both ST and snRNA-seq were performed on the same tissue block for 34 donor tissues; 17 donor tissues had only ST data, and two donor tissues had only snRNA-seq data. Additionally, a subset of tissues was stained using *in situ* and immunofluorescence techniques for validation. Overview of the processes that were performed on each tissue can be found in Table S1.

### Identifying transcriptomic changes with accurate spatial resolution using Visium ST

We performed spatially resolved transcriptome-wide sequencing on 172 tissue sections from 51 donor tissues profiled. Spots within each Visium capture array were assigned to gray matter layers 1–6 and the white matter. Anatomical annotation regions (AARs) were assigned based on the cell-type composition and density observed in our H&E-stained tissues unique to each layer (STAR Methods). After quality control (QC) and filtering (STAR Methods), our ST dataset reliably measured the expression of 13,310 genes across 671,397 ST spots (Table S2). Dimensionality reduction of all ST spots in our dataset revealed that most of the spot-level variability was attributable to our cortical-layer annotation (Figures 1B and S1A–S1D), supporting the validity and use of these annotations for data integration.

To identify differentially expressed genes (DEGs) across AARs, we used Splotch,^43^^,^^44^ a hierarchical Bayesian statistical model of spatial gene expression (STAR Methods). Barcode-capture ST methods tend to have a lower capture efficiency than snRNA-seq or bulk RNA-seq methods,^45^^,^^46^^,^^47^ causing measurements of lowly expressed genes to be disproportionately affected by dropout. To overcome this issue, along with the biological and technical sparsity of individual ST experiments, Splotch integrates information across multiple tissue sections via our manual annotations to produce posterior estimates of gene expression.

To validate whether our annotations replicated the known biology of cortical layers, we used Splotch to determine the expression of anatomical layer markers throughout our AARs. Importantly, we found that layer marker distributions within our AARs agreed with the biology described in the literature,^11^^,^^48^ with *RELN* displaying the highest level of expression in annotated layer 1, *CARTPT* in layers 2 and 3, *RORB* in layer 4, *PCP4* in layers 4 and 5, and *MOBP* in the white matter (Figure 1C).

### Identifying transcriptomic changes at single-nucleus resolution using snRNA-seq

Visium ST generates accurate spatial maps of gene expression, but its resolution is limited by the size of each ST spot. These “spots” are 55 μm in diameter with a 100-μm center-to-center distance, containing roughly 2–10 cells. Thus, to complement our spatial transcriptomic analysis, we used snRNA-seq to perform transcriptome-wide sequencing with single-nucleus resolution. Tissues used for snRNA-seq were sectioned in the same plane of the same tissue block as they were for ST experiments to capture cell types within cortical layers represented in ST experiments. We profiled tissue blocks from 36 donors and obtained transcriptomes of 117,914 nuclei after QC (Table S3). Using unbiased clustering analysis, we identified eight distinct clusters corresponding to the major cortical cell types (Figures 1D and S1E), including astrocytes, endothelial cells, excitatory neurons, inhibitory neurons, microglia, oligodendrocytes, oligodendrocyte precursor cells (OPCs), and pericytes (Figure 1E).

### Cortical layer age- and sex-associated genes

**Figure 2:**
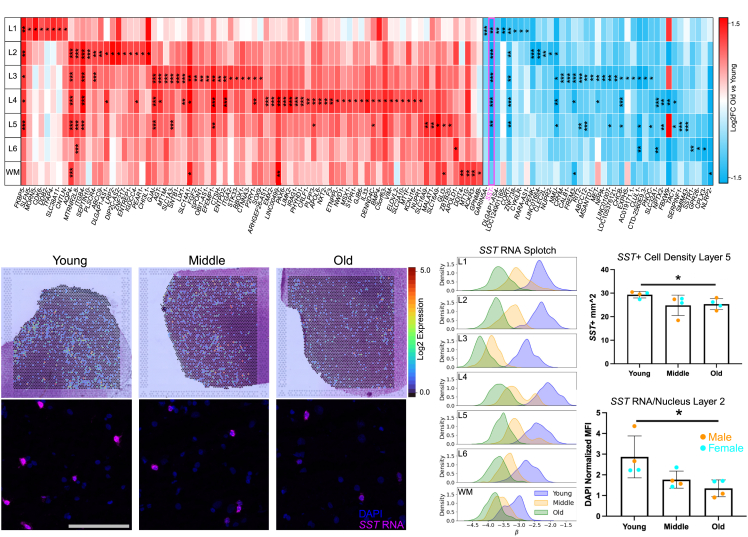
A spatial aging gene atlas of the human dlPFC (A) Heatmap displaying top ST DEGs across cortical layers between old (*n* = 21) and young (*n* = 11) donors. Scale bar represents log2FC comparison between old and young groups, where genes with a higher (lower) expression in the old cohort are shown in red (blue). Asterisks are added for significant changes in mRNA transcript by layer: ∗BF > 3 and |log2FC|>1, ∗∗BF > 10 and |log2FC|>1, ∗∗∗BF > 30 and |log2FC|>1. (B) Representative images of *SST* mRNA expression measured in log-scaled counts across representative ST arrays (top row) and visualized within *in situ*-stained tissues (bottom row) across young, middle, and old donors. The same intensity scale was used for each representative *in situ* image (scale bar, 100 μm). (C) Kernel density estimate plots displaying posterior distributions over expression rate (β) (*x* axis, log-scaled counts (β); *y* axis, probability) inferred by Splotch for *SST* across each layer (row) and age group (color code). (D and E) Quantification of *in situ* (D) *SST*+ cells per square mm in layer 5 and (E) *SST* RNA mean fluorescence intensity (MFI) normalized to DAPI per nucleus within *SST*+ cells in layer 2 of the dlPFC across young (*n* = 4), middle (*n* = 4), and old (*n* = 4) donors. Error bars represent standard deviation, and dots represent individual measurements for each donor colored by sex (see legend). Significance determined using unpaired two-sided *t* test with Welch’s correction. ∗*p* < 0.05.

To identify age- and sex-associated DEGs, we used Splotch to determine the AAR-level characteristic expression rate (β) for each gene in each cortical layer for different age (young, middle, and old) and sex (male and female) groups (Table S4). Comparing young and old donors, we identified 79 genes that strongly increased with age (Bayes factor [BF] >3 and log2 fold change [log2FC] >1) and 39 genes that strongly decreased with age (BF > 3 and log2FC < −1) in at least one cortical layer (Figure 2A).

Many of the genes whose expression increased the most with age are selectively expressed by astrocytes.^49^ We observed an increase in *AQP4* in layers 2–5 and the white matter, *GJAI* in layers 3–5, and *SOX9* in layer 3 (Figure 2A). Other well-known astrocyte markers such as *GFAP* and *ALDH1L1* did not change significantly in any layer (Table S4). Among the genes whose expression declined the most with age was *SST*, encoding for the peptide inhibitory neurotransmitter somatostatin, in layers 1–5 (Figures 2A–2C). *SST*+ interneurons play a vital role in modulating activity within the dlPFC, providing inhibitory signaling onto pyramidal neuron dendrites.^50^^,^^51^ We also observed a decline in the gene *CORT* in layers 1–5 (Figure 2A), which encodes for a neuropeptide structurally similar to *SST* called cortistatin, and a loss of the *SST* receptor gene *SSTR1* in layer 6 of the old dlPFC (Figure 2A).

We then explored the role of sex on transcript differential expression (DE). This was done by assessing: (1) each gene for sex-driven DE within each age group and cortical layer, and (2) the degree to which age-driven DE patterns were supported by one or both sexes. In the first analysis, we identified 32 genes exhibiting sex-driven DE in any age group or cortical layer, with 16 genes upregulated in male donors, and 16 in female donors (Table S4). As expected, 10 of the 16 genes with higher expression in male donors were located on the male-specific region of the Y chromosome (MSY), while *XIST* (X inactive specific transcript) was up in female tissues (Table S4). We also identified multiple DEGs with higher expression in females compared to male tissues, particularly in layer 1 of tissues from old donors, such as *CCL2*, *HSPA1B*, and *TIMP1* (Table S4). In the second analysis, we found that, of the 118 genes exhibiting significant age-driven DE in the full cohort, 37 crossed the significance threshold exclusively in one sex in at least one AAR (Figures S2A and S2B). In general, however, genes exhibited strong agreement in the magnitude and direction of age-related expression change in both sexes across all cortical layers (Pearson r >0.9; Figure S2A), indicating consistent temporal remodeling of expression independent of sex.

### Loss of *SST* in the aged gray matter

Layer-specific changes in gene expression detected by Visium can be interpreted in two ways: a change in the number of cells expressing an mRNA transcript and/or a change in the level of mRNA transcript per cell. To determine which was responsible for the observed decrease in *SST*, we performed *in situ* hybridization alongside immunostaining for SST on 12 donor tissues within the same ST cohort (Figures S3A–S3D; Table S5). Interestingly, in layers 2–5, we identified both a moderate decrease in *SST*+ cell density, which was significant in layer 5 (Figure 2D), and a more pronounced decrease in *SST* transcript per *SST*+ nucleus, which was significant in layer 2 (Figure 2E). Thus, the combination of both factors resulted in the observed decrease in *SST* identified by Splotch.

While males displayed a more visually apparent decrease in *SST* transcript per nucleus with age in the validation cohort (Figure 2E), this trend did not reach the threshold of significance when sexes were separated. Further, the decrease in *SST* observed in our higher-powered ST dataset was significant in both sexes individually in layers 1–5 of the cortex, indicating that this loss of *SST* was conserved across sexes (Figure S2C).

### Changes in the cellular composition of the aging dlPFC

Alterations in cell-type composition have been reported in the aged brain.^52^^,^^53^^,^^54^ To investigate dynamic changes in cell-type composition, we compared changes measured within our snRNA-seq dataset to inferred cell-type proportion changes within cortical layers on our ST arrays. In addition to broad class cell types (Figures S4A–S4C), we also explored changes in cell-type subclusters at a finer resolution by mapping our nuclei to a reference cortex dataset^55^ (STAR Methods). This resolved our eight classes of broad class cell types into 81 annotated subclusters (Table S3; Figure S1E), many of which have functional annotations (Figure S4D).

**Figure 3:**
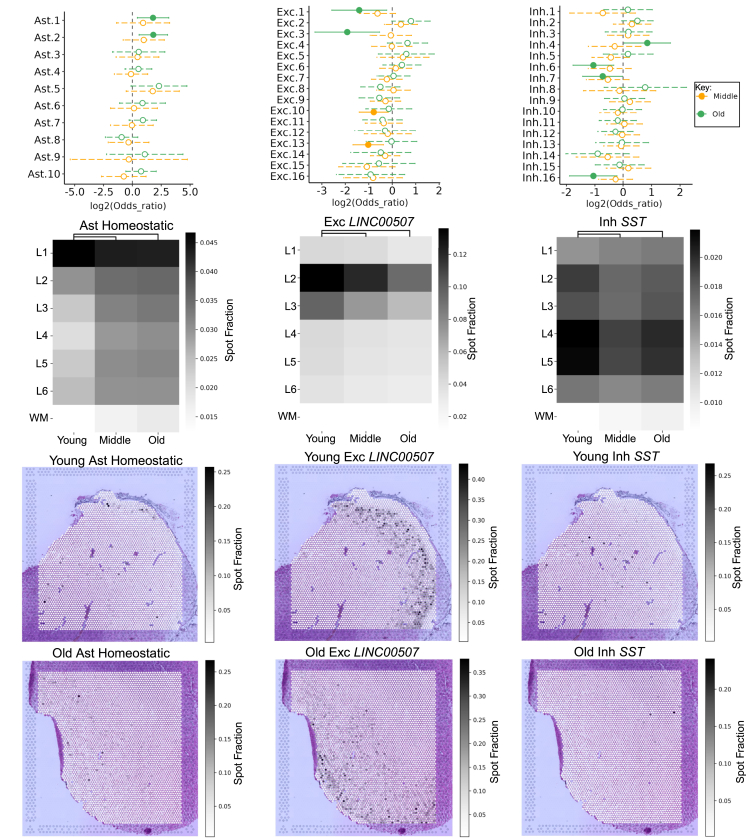
Changes in dlPFC subcell-type composition with age (A–C) MASC differential cell-type proportion testing results between young and middle (yellow) and young and old (green) groups are shown for (A) astrocyte, (B) excitatory neuron, and (C) inhibitory neuron subclusters. Odds ratios and 95% confidence intervals were calculated using MASC modeling.^56^ Points to the left (right) of the dotted line indicate decreased (increased) cell-type enrichment, respectively, in the older group. Significant results are indicated by solid, filled-in bubbles and solid interval bars (see key in C). (D–F) Heatmaps measuring abundance of (D) homeostatic astrocyte, (E) *LINC00507*+ excitatory neuron, and (F) *SST*+ inhibitory neuron merged subclusters in ST data across cortical layers predicted by cell2location. Scale bar represents predicted spot fraction of a measured cell type within each ST spot. (G–L) Visualization of predicted (G and J) homeostatic astrocyte, (H and K) *LINC00507*+ excitatory neuron, and (I and L) *SST*+ inhibitory neuron merged subclusters on young and old representative ST arrays, respectively.

Mixed-effects modeling of associations of single cells (MASC)^56^ analysis was used to determine changes in cell-type proportion between young, middle, and old donors using purely snRNA-seq data. Using MASC, we identified select classes of sub-cell types that changed with age. Astrocyte subclusters Ast. 1 and Ast. 2, which were functionally labeled as homeostatic, increased in old donors compared to young (Figure 3A). Excitatory neuron subclusters Exc. 1 and Exc. 3, which were classified as *LINC00507*+ neurons, decreased in old donors compared to young (Figure 3B). Inhibitory neuron subclusters Inh. 6 and Inh. 7, which were classified as *SST*+ neurons, decreased in old donors compared to young (Figure 3C).

We then used cell2location^57^—a deconvolution algorithm that predicts the abundance of cell types within multi-cellular ST spots using our snRNA-seq dataset as a reference—to determine the regional specificity of broad and sub-cell-type classifications, and differences in cellular composition between young, middle, and old donors across different cortical layers. Prior to running cell2location, we merged subclusters with similar annotations and discarded lowly detected subclusters, which yielded 32 merged subclusters (Table S3; STAR Methods). Marker genes of each merged cluster used for this analysis can be found in Table S6.

After performing significance testing (two-sided Welch’s *t* test, Benjamini-Hochberg false discovery rate [BH FDR] correction) at the donor level for changes in cell composition with age, we did not detect many significant differences. However, we did identify layer-specific trends in predicted abundance for the merged subclusters that recapitulated patterns from the MASC results. Homeostatic astrocyte populations (Ast. 1 and 2) were most abundant in layer 1 and displayed a decrease in average predicted spot proportion within layer 1 but an increase across all other layers with age (Figures 3D–3G and 3J). *LINC00507*+ excitatory neuron populations (Exc. 1–4) were most abundant in layers 2 and 3 and displayed a moderate decrease in average predicted spot proportion within these layers with age (Figures 3E–3H and 3K). Finally, *SST*+ inhibitory neuron populations (Inh. 1 and 5–7) were most abundant in layers 4 and 5 and displayed a moderate decrease in average predicted spot proportion within these two layers and layer 2 with age (Figures 3F–3I and 3L).

### Shared age-related gene expression programs across dlPFC broad class cell types

**Figure 4:**
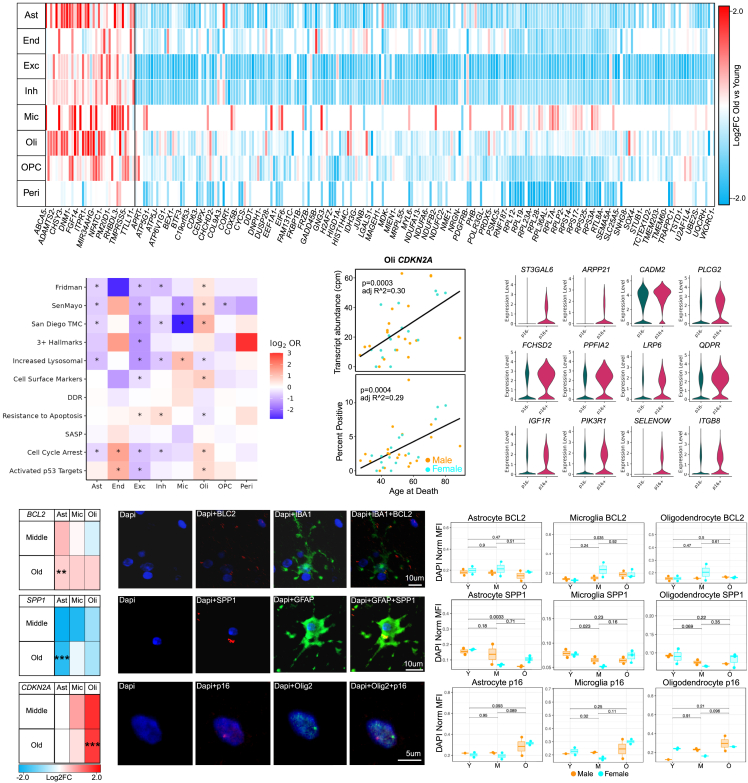
Single-nucleus and single-cell profiles of aging and senescence (A) Heatmap displaying DEGs shared across two or more broad class cell types in our snRNA-seq dataset comparing old (*n* = 12) to young (*n* = 12) donors. Scale bar represents log2FC comparison between old and young groups, where genes with a higher (lower) expression in the old cohort are shown in red (blue). Every third gene is annotated on this heatmap (full snRNA-seq DEG lists found in Table S7). BH-adjusted *p* values were derived from linear modeling with *limma-voom*. (B) Heatmap displaying differential proportion of senescent-positive nuclei between old and young cohorts for different senescence hallmarks. Cells represent test statistics from testing for difference of proportions via one-sided *t* test. Red (blue) cells indicate an increased proportion of senescent nuclei in the old (young) cohort. Reported *p* values were derived from one-way ANOVA tests on MASC models.^56^ Nominal *p* values are annotated onto cells. ∗p < 1e−2. (C) Scatterplots of average *CDKN2A* transcriptomic abundance (top) and *CDKN2A* positivity (bottom) in oligodendrocytes across donor age. *Post hoc* linear regression lines with corresponding *p* values and R^2^ values derived from linear modeling are added for clarity. Each dot represents an individual donor, colored by sex (see legend). (D) Violin plots of genes differentially expressed between *CDKN2A* (p16) negative (green) and positive (magenta) oligodendrocytes. (E–G) Heatmaps of (E) *BCL2*, (F) *SPP1*, and (G) *CDKN2A* expression among glial cell types in our snRNA-seq cohort. Scale bar represents log2FC comparison between old and young groups, where red (blue) indicates increased (decreased) expression in middle/old donors compared to young. BH-adjusted *p* values were derived from linear modeling with *limma-voom*. Nominal *p* values are annotated onto cells. ∗*p* < 0.05, ∗∗*p* < 1e−2, ∗∗∗*p* < 1e−3. (H–J) Representative images taken of (H) BCL2+ IBA1+ microglia, (I) SPP1+ GFAP+ astrocytes, and (J) p16+ OLIG2+ oligodendrocytes. DAPI depicted in blue, protein targets (BCL2, SPP1, p16) depicted in red, cell-type markers (IBA1, GFAP, OLIG2) depicted in green. Scale bars added for clarity. (K–M) Quantification of DAPI normalized MFI per cell of (K) BCL2, (L) SPP1, and (M) p16 in glial cell types across young (labeled Y; *n* = 4), middle (labeled M; *n* = 4), and old (labeled O; *n* = 5) donors. Annotated *p* values comparing measurements between age groups were derived using unpaired two-sided *t* test with Welch’s correction. Dots represent individual measurements for each donor colored by sex (see legend).

We next assessed age-related DEGs within broad class cell types profiled in our snRNA-seq dataset. We identified 612 genes that significantly increased (adjusted *p* <0.05, log2FC > 1) with age, and 1,002 genes that significantly decreased (adjusted *p* <0.05, log2FC < −1) with age in at least one broad class cell type, when comparing young and old donors (Figure S5A; Table S7). While many of these DEGs were specific to just one cell type, 254 DEGs were shared across two or more broad class cell types, with 34 increasing and 220 decreasing with age (Figure 4A). Gene Ontology (GO) annotation of these 220 shared genes decreasing with age revealed a shared loss of nuclear-residing genes functionally related to oxidative phosphorylation and cytoplasmic translation (Figures S5B and S5C; Table S8). Astrocytes, excitatory neurons, and inhibitory neurons individually displayed decreased expression in genes involved in oxidative phosphorylation, while endothelial cells, excitatory neurons, OPCs, and pericytes individually displayed decreased expression in ribosomal subunit component (RPL/RPS)-encoding genes (Table S8).

### dlPFC cell types vary in their expression of canonical senescence hallmarks

We then evaluated the expression patterns of known senescence hallmarks and curated gene sets across nuclei in our snRNA-seq dataset. To accomplish this, we utilized the SenMayo^58^ and Fridman Senescence Up lists,^59^ two widely used curated senescence gene lists, as well as a list of senescence target genes curated by the San Diego Senescence Tissue Mapping Center (SD TMC) in the SenNet consortium. These three lists include genes from multiple senescence hallmarks. We also used seven gene lists representing individual hallmarks of senescence, six of which were adapted from a recent SenNet consortium review^39^: cell-cycle arrest, SASP, DDR, resistance to apoptosis, increased lysosomal content, and cell surface markers. The final individual hallmark list we included was a comprehensive list of p53-activated gene targets,^60^ as p53 transcription factor activity drives a major growth arrest pathway that maintains senescence.^20^ A summary of the genes present within each senescence hallmark list can be found in Table S9.

Each nucleus in our snRNA-seq dataset was assigned module scores quantifying the level of expression of each senescence gene list. Nuclei were deemed positive for a given hallmark if their module score ranked above the hallmark’s threshold, which we set as the top five percentile from the module scores of the young age group (STAR Methods; Figure S6A). While excitatory neurons, inhibitory neurons, and astrocytes generally displayed a decreased proportion of nuclei positive for senescence hallmarks with age, oligodendrocytes and endothelial cells displayed an increased proportion of positive nuclei for senescence hallmarks with age (Figure 4B). Although we observed a minimal increase in the proportion of oligodendrocytes positive for three or more hallmarks at once with age, oligodendrocytes did display an increased proportion of nuclei positive for all multi-hallmark gene lists (SenMayo, Fridman, and SD TMC) and three individual hallmarks (cell surface markers, cell-cycle arrest, activated p53 targets) (Figure 4B).

In addition to assessing the DDR in our snRNA-seq dataset, we performed paired *in situ* and immunofluorescence stains to visualize telomere-associated DDR foci (TAF)^61^ in our fresh frozen dlPFC tissue. TAF events were defined as a colocalization of foci from DDR marker γH2AX and telomere foci labeled by a TelC probe (STAR Methods; Figures S7A–S7C). Interestingly, we witnessed a peak in γH2AX foci per nucleus and nuclear TAF positivity in the middle group, which then dwindled back to baseline levels in the old group (Figures S7D–S7F). This trend was especially prominent in the white matter and the border between gray and white matter (Figures S7D–S7F). This result closely mimicked the transcriptomics for oligodendrocytes, the most abundant cell type in the white matter, which displayed a significantly increased proportion of nuclei positive for the DDR in middle age compared to young, but no change in old age compared to young (Figure S6B). Previous studies have suggested that DNA damage increases with age in the human brain.^62^^,^^63^ A reduction in the DDR following middle age would potentially make older tissues more susceptible to this increased DNA damage. This relationship will need to be explored further in future experiments.

Exploring oligodendrocytes further, we identified a significant age-related increase in both transcriptomic abundance of cell-cycle arrest marker *CDKN2A* and the number of *CDKN2A*-positive oligodendrocytes (Figure 4C). We then identified genes differentially expressed between *CDKN2A*-positive and -negative oligodendrocytes (Figure 4D; Table S10). Among genes that increased in *CDKN2A*-positive oligodendrocytes was *IGF1R*, whose expression has been shown to positively correlate with senescence in the pancreas^64^ as well as genes encoding subunits of phosphatidylinositol 3-kinase (PI3K) (*PIK3R1* and *PIK3CA*), which has been linked to antiapoptotic signaling in senescent cells^39^ (Figure 4D; Table S10). Interestingly, *CDKN2A*-positive oligodendrocytes did not tend to express *CDKN1A*. In fact, coexpression of these two cell-cycle-arrest markers was rare across all dlPFC cell types (Figure S6C), which was a similar result to what had been observed in the mouse hippocampus.^65^

Overall, our analysis predicted an increased proportion of oligodendrocytes was affected by senescence hallmarks with age, accompanied by an age-related increase in *CDKN2A*-positive nuclei.

### Select senescence-related DEGs display correlated changes in their protein expression

Using Lunaphore’s COMET platform,^66^ we performed multiple cycles of immunostaining and elution on the same tissue section from multiple donors, which allowed us to visualize many dlPFC cell types and senescence targets at once (Figures S8A–S8F; STAR Methods). We performed COMET on a subset of donor tissues within our cohort (four young, four middle, five old), which allowed us to validate the expression pattern of senescence-related proteins across dlPFC cell types. We used an antibody panel that covered the major cell types in the dlPFC (astrocytes, endothelial cells, microglia, neurons, and oligodendrocytes), along with senescence-related markers that changed in our snRNA-seq dataset, such as antiapoptotic protein BCL2, secreted factor SPP1, and cell-cycle arrest marker p16 (encoded by *CDKN2A*). Similar to the results observed in our snRNA-seq dataset (Figures 4E–4G), among glial cell types (GFAP+ astrocytes, IBA1+ microglia, and OLIG2+ oligodendrocytes), we saw an age-related increase in BCL2 protein, a decrease in SPP1 protein, and an increase in p16 protein expression per cell (Figures 4H–4M; Table S11). Increase in BCL2 was significant among astrocytes in our snRNA-seq dataset and microglia in our COMET dataset (Figures 4E–4K). Decrease in SPP1 was significant in astrocytes in both our snRNA-seq and COMET dataset (Figures 4F and 4L). Finally, *CDKN2A* significantly increased in oligodendrocytes in our snRNA-seq dataset, while an increase in p16 neared the threshold of significance in astrocytes in our COMET dataset (Figures 4G and 4M).

### Pairwise spatial gene coexpression analysis reveals spatial gene expression programs strongly associated with aging in the dlPFC

**Figure 5:**
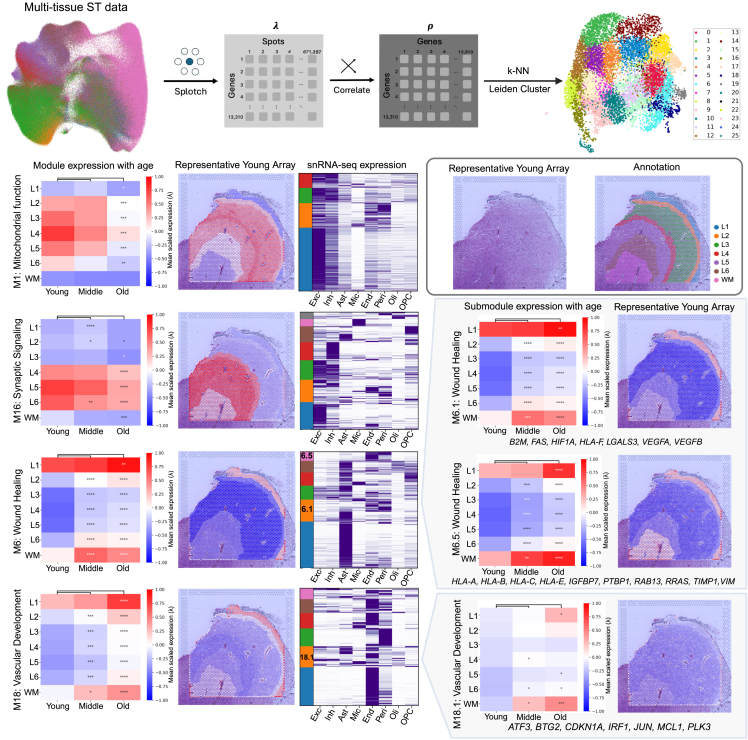
Spatially coordinated aging and senescence-related gene programs in the dlPFC (A) A spot-level coexpression analysis was applied to our spatial data, resulting in the generation of 26 unique spatial modules (STAR Methods). (B) Raw H&E of representative young ST array (left) and corresponding layer annotation (right). (C–F) Heatmap showcasing mean expression level of (C) M1, (D) M16, (E) M6, and (F) M18 by cortical layer and age group, with differential testing between middle/old versus young (left). Spot-level expression of each module on a representative young ST array (middle) utilizing the same scale as the module heatmap (left). Heatmap showcasing module gene expression (minimum-maximum scaled) across broad class cell types in our snRNA-seq dataset (right), with different colored blocks on the left side of the heatmap representing cell-type-specific submodules within that module. Submodules M6.1, M6.5, and M18.1 annotated on the left side of corresponding snRNA-seq expression heatmap (right). (G–I) Heatmap showcasing mean expression level of (G) M6.1, (H) M6.5, and (I) M18.1 by cortical layer and age group, with differential testing between middle/old versus young (left). Spot-level expression of each submodule on a representative young ST array (right) utilizing the same scale as the submodule heatmap (left). Senescence-related genes contained within each submodule (bottom). For all modules and submodules, significant changes in scores across age groups for each layer were determined using Welch’s *t* test with BH FDR correction. Asterisks indicate level of significance: ∗∗∗∗*p* < 1e−4, ∗∗∗*p* < 1e−3, ∗∗*p* < 1e−2, ∗*p* <0 .05.

Having identified individual genes that changed with age in our ST dataset, we then sought to uncover groups of genes with coordinated spatiotemporal patterns of expression in the aged dlPFC. To identify such spatiotemporal patterns of gene expression, we applied a spot-level coexpression analysis to our spatial data (Figure 5A; STAR Methods). In brief, we measured the expression of 13,310 genes across the 671,397 spots in our ST dataset and generated a Pearson correlation matrix that quantified gene-gene expression covariance on the spot level. This matrix informed a gene-gene k-nearest neighbors (K-NN) graph embedding, which was used to map genes in two-dimensional uniform manifold approximation and projection (UMAP) space and also used to cluster them into distinct modules using a Leiden algorithm. In doing so, we identified 26 unique spatiotemporal gene expression modules (Figure 5A) with each of the 13,310 genes assigned to one module (Table S12). The expression pattern of each module was measured across each cortical layer and age grouping (Figure S9A), and modules of interest were visualized on representative ST arrays (Figures 5B–5I; Figure S9B).

### Select spatial modules tied to neuronal health and function decrease with age

Among the 26 generated modules, module 1 (M1) was strongly expressed in layers 3–5 of the dlPFC and significantly decreased throughout layers 1–6 with age (Figure 5C). GO analysis revealed that this module contains genes strongly linked to mitochondrial functions, such as oxidative phosphorylation, mitochondrial translation, and electron transport chain (ETC) component assembly (Table S13). M1 genes were expressed highly in excitatory neurons in our snRNA-seq dataset (Figure 5C). Further, 38 nuclear genes under the GO term for oxidative phosphorylation were both present in M1 and significantly decreased in excitatory neurons with age in our snRNA-seq dataset (Figure S5D). Overall, decline in mitochondrial function genes, both at the single-nucleus and spatial level, potentially represents an important hallmark of aging in the dlPFC.

Module M16 was enriched in layers 4–6 of the dlPFC and significantly decreased throughout the gray matter with age (Figure 5D). GO analysis determined genes within M16 were functionally related to synaptic signaling (Table S13). M16 was highly expressed in excitatory neurons and inhibitory neurons in our snRNA-seq dataset (Figure 5D) and contains several gene markers of deep-cortical-layer neuronal subpopulations, such as *FEZF2*, *SST*, and *NPY* (Table S12). Thus, M16 shows a disruption in synaptic signaling machinery among layer 4–6 neuronal populations with age.

### Select spatial modules tied to astrocyte function and the vasculature increase with age and are enriched for senescence-associated genes

We next investigated spatially coordinated patterns of senescence-related gene expression. We were interested in uncovering gene expression patterns encoding communities of senescent cells specific to subregions of the dlPFC. To accomplish this, spatial modules were further subdivided into submodules based on the cell-type-specific expression of each gene as assessed by our snRNA-seq dataset (STAR Methods; Table S12).

Module M6 was enriched in layer 1 and the white matter of the dlPFC and significantly increased throughout every layer of the cortex with age (Figure 5E). GO analysis determined M6 to be functionally related to wound healing (Table S13). M6 was highly expressed in astrocytes in our snRNA-seq dataset (Figure 5E) and contains several astrocyte gene markers, such as *GLUL*, *ALDH1L1*, and *AQP4* (Table S12). M6.1 is a submodule within M6 that was enriched for genes highly expressed in astrocytes and vascular cells (endothelial cell and pericytes; Figure 5E) and displayed a similar spatiotemporal pattern to M6 (Figure 5G). M6.1 contained several senescence-related genes such as cell surface marker genes *B2M* and *HLA-F*; SASP-related genes *FAS*, *VEGFA*, and *VEGFB*; apoptotic resistance gene *HIF1A*; and lysosome stress gene *LGALS3* (Figure 5G; Table S9). Similarly, M6.5 is a submodule within M6 that was enriched for genes highly expressed in vascular cells (Figure 5E) and displayed a similar spatiotemporal pattern to M6 (Figure 5H). M6.5 contained senescence-related genes such as cell surface marker genes *HLA-A*, *HLA-B*, *HLA-C*, and *HLA-E*; SASP-related genes *IGFBP7*, *PTBP1*, and *TIMP1*; and genes within the Fridman senescence up list such as *RAB13*, *RRAS*, and *VIM* (Figure 5H; Table S9).

Similar to M6, module M18 was enriched in layer 1 and the white matter of the dlPFC and significantly increased throughout every layer of the cortex with age (Figure 5F). Distinct to M18, however, GO analysis determined this module to be involved in vascular development (Table S13), and M18 genes were highly expressed by vascular cells (Figure 5F), containing endothelial cell markers *CLDN5*, *PECAM1*, and *CD34* and pericyte marker *PDGFRB* (Table S12). M18.1, a submodule of M18, displayed a mixed gene expression pattern spanning multiple broad class cell types, with significant contributions made by endothelial cells and astrocytes (Figure 5F). Interestingly, M18.1 was lowly expressed throughout all layers of the young dlPFC but increased dramatically in the white matter with age (Figure 5I). M18.1 also contains senescence-related genes such as cell-cycle-arrest marker *CDKN1A;* p53-activated target genes *ATF3*, *BTG2*, and *PLK3*; antiapoptotic gene *MCL1*; and senescence-related transcription factors *JUN* and *IRF1* (Figure 5I; Table S9).

In summary, we identified spatial submodules M6.1, M6.5, and M18.1, which increased strongly with age and were enriched for senescence-related genes. While M6.1 and M6.5 were strongly expressed in layer 1 and in the white matter, M18.1 was almost exclusively expressed in the aged white matter of the dlPFC. These submodules potentially represent senescent cell sub-type profiles within the healthy aging dlPFC.

### Neighborhood analysis of senescence-related gene modules

**Figure 6:**
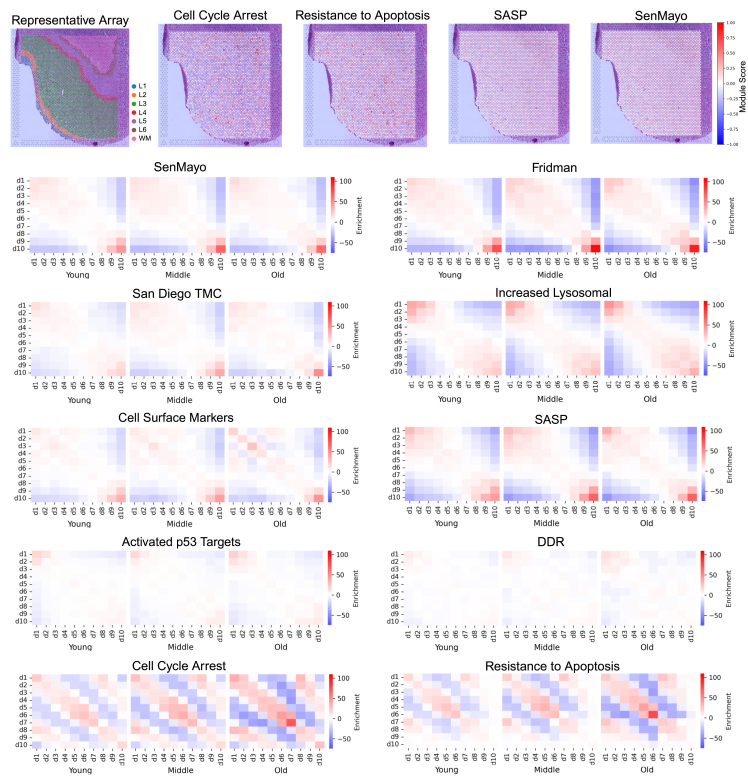
Local distribution of senescence modules with age (A) (From left to right) Representative annotated old donor tissue array alongside maps of measured senescence hallmark expression for cell-cycle arrest, resistance to apoptosis, SASP, and SenMayo on this same section. (B–K) Spatial transcriptomic spots were scored for expression of each curated multi-hallmark list from the literature (B–D) and single senescent hallmark (E–K). Module scores across ST spots were divided into deciles. For each curated senescence gene module, we display heatmaps showing spatial enrichment statistics for each decile and quantified self-enrichment.

Our spatial analysis revealed changes in expression at the level of cortical layers. To examine changes at higher spatial resolution, we focused on local, spot-level neighborhoods. We selected the 10 curated senescence-hallmark gene lists described previously (Figure 4B; Table S9). For each gene list, each ST spot in our dataset was assigned an expression score between 1 (lowest decile) and 10 (highest decile), and the “enrichment”—the propensity of spots with a given score to neighbor spots with another score—was calculated for all pairs of scores, separately for each age group (STAR Methods). We were interested in whether ST spots that scored highly for a senescence hallmark would tend to co-localize or would be more dispersed (Figures 6B–6K). Additionally, we examined the distribution of spots per expression decile in each cortical layer.

From our spatial-enrichment analysis (STAR Methods), most gene sets exhibited spatial coherence: spots with the same or similar expression score tended to neighbor each other. Visually, this is reflected by a strong diagonal signal on the pairwise enrichment plot. Senescence multi-hallmark lists curated from the literature, such as SenMayo, Fridman Senescence Up, and the SD TMC list, displayed high levels of spatial coherence (Figures 6B–6D), indicating that spots positive for senescence signatures tended to neighbor each other. Interestingly, the highest-scoring spots (d10) from these multi-hallmark lists tended to be expressed in the white matter (Figures 6A and S10A). This implicates the white matter as a potential site of senescent cell induction in the dlPFC.

Similar to the multi-hallmark lists, SASP signaling, cell surface markers, and increased lysosomal genes displayed a high degree of spatial coherence (Figures 6E–6G). High-scoring spots for the SASP and cell surface markers also displayed regional preference for the white matter (Figures 6A and S10A). Activated p53 targets and the DDR signals were more dispersed, with low- and high-scoring spots neighboring each other at comparable rates to similarly scored spots (Figures 6H and 6I). These hallmarks also displayed lower module score variability when compared to other senescent hallmarks, indicating greater homogeneity in expression (Figure S10B). Cell-cycle arrest and resistance to apoptosis displayed a high degree of spatial coherence (Figures 6J and 6K) but also showed local neighborhoods of high-expressing spots among pervasive low expression of these modules (Figure 6A). This was reflected in their spatial-enrichment plots, which displayed two “off-diagonals” from the center (Figures 6J and 6K). This pattern of interleaving regions of high and low expression may be explained by the mixture of sporadic stressors and paracrine signaling processes (e.g., SASP^24^) controlling cell-cycle arrest. High-scoring spots for cell-cycle arrest also displayed a regional preference for the white matter (Figures 6A and S10A).

## Discussion

A hallmark of the aging dlPFC identified in this study was a sharp decline in *SST* transcript and a moderate loss in *SST*+ neuron density, both of which have been implicated in various neurological diseases and disorders. Loss of *SST* transcript and *SST*-related synaptic deficits have been identified in the dlPFC of patients suffering from schizophrenia.^67^^,^^68^^,^^69^ In our previous work, we found that *SST* transcript was reduced in gray-matter layers 1–6 comparing ALS-FTD patients with and without cognitive impairment to nonpathological controls.^70^ Loss of *SST*+ interneurons has been observed in the human dlPFC during the early stages of Alzheimer’s disease^71^ and was correlated with a decline in episodic memory, perceptual orientation, and semantic memory.^72^ As such, this loss of *SST* transcript and *SST*+ cells we observe in the healthy aging dlPFC potentially progresses further into early-stage disease and cognitive impairment later in life.

We observed a strong decline in nuclear genes tied to mitochondrial functions in both our ST and snRNA-seq datasets. Mitochondrial dysfunction is a known hallmark of the aging brain^73^^,^^74^ and neurodegenerative disorders.^75^^,^^76^^,^^77^ In neurons, mitochondria generate ATP through oxidative phosphorylation in order to sustain synaptic transmission and cell maintenance/repair.^73^^,^^75^ Experiments isolating mitochondria from brain tissue of animal models of aging and disease revealed deficits in ETC function when compared to young healthy animals.^78^^,^^79^ Complex 1 activity in particular significantly decreased in mitochondria isolated from the aged mouse cortex when compared to young controls.^80^ Proper ETC component assembly relies on coordinated activity between nuclear and mitochondrial encoded subunit genes.^81^ As such, this strong decline in nuclear-residing mitochondrial genes we observe in the aged dlPFC may be directly tied to the decline in mitochondrial function we see in animal models of aging.

Although RPL/RPS genes significantly decreased among multiple cell types in our snRNA-seq dataset, spatial module M14, which was functionally tied to cytoplasmic translation (Table S13), did not significantly decrease with age (Figure S9A). Contrasting with the loss of genes related to mitochondrial function, decline in ribosomal subunit genes with age might be specific to the nucleus, while cytoplasmic expression is relatively unaffected.

Mature astrocytes re-enter the cell cycle in response to tissue damage, such as stroke, inflammation, and neurodegenerative disease pathology.^82^ Our study identified an increased proportion of homeostatic astrocytes and increased expression of astrocyte markers in select regions of the aged cortex. While certain astrocyte-related genes increased across specific aged cortical layers (*AQP4*, *GJAI*, *SOX9*), others did not change (*ALDH1L1*, *GFAP*). These results suggest there was not an increase in all astrocytes in the dlPFC. Instead, specific regional populations of astrocytes increased in abundance with age, which supports previous studies suggesting that astrocytes display region-specific gene expression programs in the brain.^83^^,^^84^

We identified a coordinated spatial gene expression program related to wound healing, M6, which was expressed by astrocytes and endothelial cells in layer 1 and the white matter, and significantly increased throughout the cortex with age. M18, related to vascular development, contained vascular cell-related genes and displayed a similar spatiotemporal pattern to M6. Astrocytes communicate with endothelial cells and pericytes at the blood-brain barrier (BBB) through their endfeet,^85^ which wrap along the surface of the endothelium,^86^ and contribute to BBB structure. Interestingly, M6 contains pro-angiogenic growth factors such as *AGT*, *ANGPTL4*, *FGF2*, *VEGFA*, and *VEGFB* (Table S12), of which *AGT* and *FGF2* display significantly increased expression in layer 3 with old age (Figure 2A). Astrocytes have been shown to secrete each of the above pro-angiogenic growth factors in aging and disease, influencing BBB development, maintenance, and permeability.^87^^,^^88^^,^^89^^,^^90^ It is possible that the age-related changes in vascular development we observed were influenced by interactions with astrocytes in the dlPFC. In the future, we would like to confirm at subcellular resolution whether cells expressing these gene programs lie near one another locally and/or are more abundant in the aged cortex.

Our results implicate the white matter as a hotspot for senescent cell induction in the dlPFC. The white matter is selectively vulnerable to the aging process,^91^^,^^92^ and many studies have reported a similar selective incidence of senescence in white matter cell types of the aged brain in animal models.^93^^,^^94^^,^^95^^,^^96^ While primary senescence is induced by internal stressors, such as DNA damage and oxidative stress, secondary senescence is induced by external stimuli, such as paracrine SASP factors. Given that the non-neuronal cell types with a higher propensity to adopt a senescent state reside in the white matter, and SASP signaling is also elevated in the white matter relative to other regions, such a positive-feedback loop is likely part of the reason the white matter of the dlPFC is selectively vulnerable to senescence.

Spatial submodules M6.1, M6.5, and M18.1 represent candidate senescence-related gene programs in the aged cortex. By exploring these submodules for individual genes that significantly changed with age, we can identify novel markers of senescence in the dlPFC. *EFEMP1* was present within module 6.1 and significantly increased in layers 3–5 with age (Figure 2A). *EFEMP1* encodes for a secreted protein belonging to the fibulin family, which regulates extracellular matrix structure and function and promotes angiogenesis.^97^ *VIM* (encoding for vimentin) was present within module 6.5 and significantly increased in layer 4 with age (Figure 2A). Vimentin is an extracellular matrix component secreted in response to inflammation at sites of immune cell recruitment,^98^ and overproduction of vimentin induces a senescence-like morphology in cultured human fibroblasts.^99^ Finally, *DUSP1* was present within M18.1 and increased in the white matter with age, nearing the threshold of significance (BF = 3.4, log2FC = 0.94; Table S4). *DUSP1* is a phosphatase that can inhibit the induction of cell death^100^ and plays a role in T cell activation.^101^ More work needs to be done to validate the importance of these genes to senescence in the dlPFC.

### Limitations of the study

A major limitation of human tissue-profiling studies is being able to amass a robust age and sex diverse cohort. We adopted age brackets to maximize parity in group size and sex representation: young (<45 years old), middle (45–55 years old), and old (>55 years old).

Due to the difficulty of dissociating intact viable single cells from brain tissues, snRNA-seq is often the preferred method for achieving single-nucleus resolution of the transcriptome.^102^ However, single-nucleus isolation often has a representation bias favoring neuronal populations while neglecting microglial and vascular populations. Most microglia we isolated were annotated as surveilling (*n* = 3613 nuclei) with the remainder belonging to other specialized microglial cell types (*n* = 784 nuclei). We are underpowered to identify age- or sex-related changes in rare cell-type subpopulations within these cell types in our snRNA-seq dataset.

No one marker can be used to identify a senescent cell. In Figures 4H–4M, we display results from a pilot COMET run that identified changes in individual senescence-associated markers with age that mimicked trends we observed in our snRNA-seq dataset. In the future, we would like to expand both our cohort of tissues and panel of senescence markers profiled by COMET to identify and score cells positive for multiple senescence hallmarks at once and to determine subcellular spatial patterning of senescence phenotypes in the dlPFC. Additionally, while integrating with disease datasets is beyond the present scope, we would like to compare senescence signatures in healthy aging to dlPFC tissue afflicted by pathology, such as Alzheimer’s disease and frontotemporal dementia.

## Resource availability

### Lead contact

Further information and requests for resources and reagents should be directed to and will be fulfilled by the lead contact, Hemali Phatnani (hp2286@cumc.columbia.edu).

### Materials availability

This study did not generate new unique reagents.

### Data and code availability

• Raw sequencing data is publicly available on the SenNet consortium portal https://dx.doi.org/10.60586/SNT456.VHRV.853 and dbGAP under the study ID phs004428.v1.
• Summary single-nucleus annotated dataset is provided on Zenodo: https://doi.org/10.5281/zenodo.17467925.
• Code used in this paper is available on GitHub: https://github.com/JasonMares63/Columbia-TMC-DLFPC-Senescence/tree/main https://doi.org/10.5281/zenodo.17860354.

## Acknowledgments

Research reported in this publication was supported by the 10.13039/100000049National Institute on Aging of the 10.13039/100000002NIH under award number U54AG076040. We are grateful to the San Diego TMC for allowing us to use their group’s senescence gene list. We thank the tissue donors whose generosity enabled this study. Graphical abstract and Figure 1A were created using BioRender.com.

## Author contributions

Conceptualization, V.M. and H.P.; methodology, N.X.S., J.M., A.C.D., V.M., and H.P.; investigation, N.X.S., S.G., I.H., N.B., O.C., K.K., S.K., K.H., and B.T.F.; formal analysis, N.X.S., J.M., A.C.D., I.H., C.A.J., J.E., and B.G.; data upload, C.M. and V.M.; writing, N.X.S., J.M., A.C.D., C.A.J., I.H., J.P., V.M., and H.P.; writing – review & editing, all authors; resources, A.F.T., H.R., C.P.S., V.M., and H.P.; supervision, Y.S., V.M., and H.P.; funding acquisition, A.F.T., H.R., C.P.S., Y.S., V.M., and H.P.

## Declaration of interests

The authors declare no competing interests.

## STAR★Methods

### Key resources table

**Table:** 

REAGENT or RESOURCE	SOURCE	IDENTIFIER
**Antibodies and probes**		

Alexa Fluor 488 Donkey Anti-Chicken	Thermo Fisher Scientific	Cat# A78948; RRID: AB_2921070
Alexa Fluor 488 Donkey Anti-Goat	Thermo Fisher Scientific	Cat# A-11055; RRID: AB_2534102
Alexa Fluor 568 Donkey Anti-Mouse	Thermo Fisher Scientific	Cat# A10037; RRID: AB_11180865
Alexa Fluor 647 Donkey Anti-Rabbit	Thermo Fisher Scientific	Cat# A31573; RRID: AB_2536183
BCL2	Cell Signaling Technology	Cat# 15071; RRID: AB_2744528
CD31	R&D Systems	Cat# AF3628; RRID: AB_2161028
DAPI	Lunaphore Technologies	Cat#DR100
GFAP	Novus	Cat# NBP1-05198; RRID: AB_1556315
***γ***H2AX SER139	Cell Signaling Technology	Cat# 2577; RRID: AB_2118010
IBA1	Fujifilm Wako	Cat# 011-27991; RRID: AB_2935833
Map2	Abcam	Cat# AB5392; RRID: AB_2138153
Olig2	R&D Systems	Cat# AF2418; RRID: AB_2157554
p16	Abclonal	Cat# A11651; RRID: AB_2861619
SPP1	Santa Cruz Biotechnology	Cat# SC-21742; RRID: AB_2194997
SST	Atlas Antibodies	Cat# HPA019472; RRID: AB_1857360
*SST* RNAscope C1 probe	Advanced Cell Diagnostics	Cat# 310591
TelC-647	PNA Bio	Cat# F1013

**Biological samples**		

Healthy Adult BA9 and BA46 Tissue	University of Edinburgh Sudden Death Brain Bank and New York Brain Bank (NYBB)	See Table S1 “Edinburgh/NYBB ID” Column

**Chemicals, peptides, and recombinant proteins**		

BSA	NEB	Cat# B9200S
CaCl2	Millipore Sigma	Cat# 21115
Dewax and HIER Buffer H	Epredia	Cat# TA-999-DHBH
DTT	Millipore Sigma	Cat# 646563
EDTA pH 8.0	Thermo Fisher	Cat# AM9260G
Elution Buffer	Advanced Cell Diagnostics Inc.	Cat# BU07-L
Ethanol (EtOH)	Sigma Aldrich	Cat# E7023
Formamide	Sigma Aldrich	Cat# F7503
Imaging Buffer	Advanced Cell Diagnostics Inc.	Cat# BU09
IPEGAL CA-630	Millipore Sigma	Cat# I8896
Magnesium Acetate	Millipore Sigma	Cat# 63052
MACS buffer (10% BSA)	Miltenyi Biotec	Cat# 130-091-376
Multistaining Buffer	Advanced Cell Diagnostics Inc.	Cat# BU06
Nuclei EZ Lysis Buffer	Sigma	Cat# NUC101-KT
OCT	Fisher Healthcare	Cat# 4585
OptiPrep	Stemcell Technologies	Cat# 07820
Paraformaldehyde (16%)	Electron Microscopy Sciences	Cat# 15710
Prolong Gold Fluoromount with DAPI	Cell Signaling Technology	Cat# 8961S
Quenching Buffer	Advanced Cell Diagnostics Inc.	Cat#BU08-L
Recombinant Albumin	NEB	Cat# B9200S
RNAse Inhibitor	Millipore Sigma	Cat# 03335402001
RNAse Inhibitor	Takara	Cat# 2313B
Salmon Sperm DNA	Rockland	Cat# MB-103-0025
Tris HCl pH 7.0	Thermo Fisher	Cat# AM9850G
Tris HCl pH 7.4	Boston BioProducts	Cat# BBT-74
Tris HCl pH 8.0	Thermo Fisher	Cat# AM9855G
Tween 20	Sigma Aldrich	Cat# P9416
20X SSC Buffer	EMD Millipore	Cat# 8310

**Critical commercial assays**		

Agilent High Sensitivity DNA Kit	Agilent	Cat# 5067-4626
Chromium Next GEM Chip J Single Cell Kit	10x Genomics	PN-1000234
Chromium Next GEM Single Cell Multiome ATAC + Gene Expression Reagent Bundle	10x Genomics	PN-1000283
Dual Index Kit TT Set A	10x Genomics	PN-1000215
KAPA Library Quantification Kit for Illumina Platforms	KAPA Biosystems	Cat# KK4824
Library Construction Kit	10x Genomics	PN-1000190
NovaSeq 6000 S4 Reagent v1.5 Kit (200 Cycles)	Illumina	Cat# 20028313
NovaSeq X 25B Reagent Kit (300 Cycles)	Illumina	Cat# 20104706
RNAscope Multiplex Fluorescent Reagent Kit v2 with TSA Vivid Dyes	Advanced Cell Diagnostics	Cat# 323270
Single Index Kit N Set A	10x Genomics	PN-1000212
Visium Spatial Gene Expression Slide and Reagent Kit	10x Genomics	PN-1000184
Visium Spatial Tissue Optimization Slide and Reagent Kit	10x Genomics	PN-1000193
10X Genomics’ Chromium Nuclei Isolation Kit	10x Genomics	PN-1000493

**Deposited data**		

Code used for Visium ST and snRNA-seq analyses	This Paper	https://github.com/JasonMares63/Columbia-TMC-DLFPC-Senescence/tree/main https://doi.org/10.5281/zenodo.17860354
GRCh38 2020	10x Genomics	NA
Published snRNA-seq dataset	Green et al.^55^	https://www.synapse.org/Synapse:syn31512863
Raw and Analyzed Data	This Paper	https://dx.doi.org/10.60586/SNT456.VHRV.853dbGAP: phs004428.v

**Software and algorithms**		

Bamboozle	Pinder et al.^103^	https://github.com/sandberg-lab/dataprivacy
Cellprofiler 4.2.8	The Broad Institute	https://cellprofiler.org/; RRID: SCR_007358
Cell2location	Kleshchevnikov et al.^57^	https://github.com/BayraktarLab/cell2location; RRID: SCR_024859
DoubletFinder	McGinnis et al.^104^	chris-mcginnis-ucsf/DoubletFinder: R package for detecting doublets in single-cell RNA sequencing data; RRID: SCR_018771
HALO	Indica Labs	RRID: SCR_018350
Harmony	Korsunsky et al.^105^	immunogenomics/harmony: Fast, sensitive and accurate integration of single-cell data with Harmony; RRID: SCR_022206
HORIZON Viewer	Lunaphore Technologies	https://lunaphore.com/news/lunaphore-commercially-launches-horizon-software-to-support-comet-hyperplex-image-analysis/
edgeR v.3.36.0	PMCID: PMC2796818	RRID: SCR_012802
limma v.3.36.0	ISSN: 1474-760X	https://portal.issn.org/resource/ISSN/1474-760X; RRID: SCR_010943
Loupe Browser Version 6.3.0	10X Genomics	RRID: SCR_018555
MapMyCells	Brain Knowledge Platform	https://portal.brain-map.org/atlases-and-data/bkp/mapmycells; RRID: SCR_024672
MASC	Fonseka et al.^56^	https://github.com/immunogenomics/masc; RRID: SCR_025632
Metascape v3.5.20250701	Zhou et al.^106^	https://metascape.org/gp/index.html#/main/step1; RRID: SCR_016620
QuPath 0.5.1	https://doi.org/10.1038/s41598-017-17204-5	https://qupath.github.io/; RRID: SCR_018257
Seurat v5.1.0.	Satjita Lab	https://satijalab.org/seurat/articles/install_v5.html; RRID: SCR_007322
Space Ranger v.2.0.0	10X Genomics	RRID: SCR_025848
Splotch	Äijö et al.^43^	https://zenodo.org/record/2566612
Squidpy	Palla et al.^107^	https://squidpy.readthedocs.io/en/stable/; RRID: SCR_026157
TANGRAM	Biancalini et al.^108^	https://github.com/broadinstitute/Tangram

### Experimental model and study participant details

#### Postmortem tissue

dlPFC tissue (Brodmann areas 9 and 46) from sudden-death non-neurological control brains was acquired from the University of Edinburgh Sudden Death Brain Bank and the New York Brain Bank (NYBB) at Columbia University. Informed consent was acquired by the University of Edinburgh and Columbia University through their own respective institutional review board (IRB) protocols and samples were transferred to the Columbia University Tissue Mapping Center (CUSTMAP) in accordance with all applicable foreign, domestic, federal, state, and local laws and regulations for processing, sequencing and analysis. In depth subject metadata can be found in Table S1.

### Method details

#### Tissue sectioning

Region BA9 or BA46 of the dlPFC was identified at autopsy, blocked out, and flash frozen on nitrogen vapors.^109^ Frozen dlPFC tissue blocks were oriented perpendicular to the pial surface and trimmed to create sampling regions approximately 7 mm × 7 mm in size spanning the full gray matter from the pial surface to the white matter. Tissue blocks were required to have a balance of both gray and white matter to proceed with downstream experiments. Trimmed blocks were embedded in Tissue Plus OCT Compound (Fisher Healthcare; #4585) and cryosectioned at −17°C.

For Visium ST, sections of 10 μm thickness cut perpendicular to the pial surface were collected onto prechilled Visium Spatial Gene Expression Slides (10x Genomics; PN-1000185). Four technical replicates for each donor were collected across two Visium slides to minimize technical batch effects. Technical replicates on the same Visium slide are approximately 30–50 μm apart. Where necessary due to tissue sectioning artifacts, additional technical replicates were collected and processed on a later date.

For snRNA-seq, 5-10 sections of 50 μm thickness were cut from the same tissue block in the exact same plane that sections were cut for Visium ST experiments. This was to ensure we captured the same balance of cell types and cortical layers across both techniques for each donor. We then proceeded to the nuclei isolation protocol.

For TAF and *SST* RNAscope/IF experiments, sections of 10 μm thickness were collected on prechilled superfrost plus slides. 1 tissue section was collected for each donor, with 4 dlPFC samples analyzed for each age group (See Table S1).

For Comet experiments, sections of 6 μm thickness were collected from FFPE tissues onto superfrost plus slides. 1 tissue section was collected for each donor, with 3 dlPFC samples analyzed for each age group (See Table S1).

#### Nuclei isolation protocol

Different nuclei isolation techniques were used for subsets of the 37 donor tissues profiled for snRNA-seq (Table S1). Importantly, for all samples following nuclei isolation, single-nucleus RNA/ATAC sequencing was performed according to the 10X Genomics’ Chromium Next GEM Single Cell Multiome ATAC + Gene Expression User Guide (CG000338 Rev F).

Nuclei were isolated from 20 frozen postmortem dlPFC samples using a protocol previously published by Chatila et al. (2023).^110^ To profile nuclei from the same representative plane used in our Visium experiments, approximately 5–10 50 μm cryosections of postmortem tissue were collected into pre-chilled 2 mL Eppendorf tubes. On ice, each sample received 2 mL of Nuclei EZ Lysis Buffer (Sigma; #NUC101-KT) and was transferred to dounce tissue grinders (DWK Life Sciences; #357538). Samples were homogenized using ∼20–25 strokes with the loose pestle followed by ∼20–25 strokes with the tight pestle. The homogenized samples were transferred to a clean, pre-chilled 15 mL Falcon tube, where they received an additional 2 mL of Nuclei EZ Lysis Buffer. After pipette mixing, samples were incubated on ice for 5 min. Samples were centrifuged at 4C for 5 min at 500g. After removing the supernatants, nuclei pellets were resuspended in 4 mL of Nuclei EZ Lysis Buffer and incubated on ice for 5 min. Following a second centrifugation at 4C for 5 min at 500g, the supernatants were removed, and the nuclei pellets were resuspended in 1 mL of Nuclei Suspension Buffer (NSB: PBS, 0.01% BSA (NEB; #B9200S), 0.1% RNAse inhibitor (Takara; #2313B)). The samples were centrifuged once more at 4C for 5 min at 500g. The nuclei pellets were resuspended in NSB, filtered through a 35 μm strainer, and counted. Each nuclei suspension was diluted for a targeted nuclei recovery of 5,000 nuclei per sample.

Nuclei were isolated from 14 frozen postmortem dlPFC samples using a previously published protocol.^111^^,^^112^ In brief, approximately 5–10 50 μm cryosections of postmortem tissue were collected into pre-chilled 2 mL Eppendorf tubes. Sections were then transferred to a pre-chilled Dounce homogenizer containing 700 μL of pre-chilled homogenization buffer (320 mM sucrose, 5 mM CaCl2 (Millipore Sigma; #21115), 3 mM magnesium acetate (Millipore Sigma; #63052), 10 mM Tris HCl pH 7.8 (Thermo Fisher; #AM9850G and #AM9855G), 0.1 mM EDTA pH 8.0 (Thermo Fisher; #AM9260G), 0.1% IGEPAL CA-630 (Millipore Sigma; #I8896), 1 mM DTT (Millipore Sigma; #646563), and 0.4 U/μL Protector RNase inhibitor (Millipore Sigma; #03335402001)). Samples were then homogenized using 10 strokes of a loose pestle, followed by 10–15 strokes of a tight pestle. Following a 5 min incubation, homogenized samples were strained through a 70-μm Flowmi Cell Strainer (Bel-Art; #H13680-0070). Strained samples were then combined with an equal volume of working solution (50% OptiPrep (STEMCELL Technologies; #07820), 5 mM CaCl2, 3 mM magnesium acetate, 10 mM Tris HCl pH 7.8, 0.1 mM EDTA pH 8.0, and 1 mM DTT). Using a 15-mL conical tube, this mixture was then layered onto 750 μL of density gradient 1 (30% OptiPrep, 134 mM sucrose, 5 mM CaCl2, 3 mM magnesium acetate, 10 mM Tris HCl pH 7.8, 0.1 mM EDTA pH 8.0, 1 mM DTT, 0.04% IGEPAL CA-630, and 0.17 U μl−1 RNase inhibitor), which was in turn layered over 300 μL of density gradient 2 (40% OptiPrep, 96 mM sucrose, 5 mM CaCl2, 3 mM magnesium acetate, 10 mM Tris HCl pH 7.8, 0.1 mM EDTA pH 8.0, 1 mM DTT, 0.03% IGEPAL CA-630, and 0.12 U μl−1 RNase inhibitor). Nuclei were separated by centrifugation at 3,000g for 20 min at 4°C using a pre-chilled swinging-bucket rotor with the brake off. Approximately 150 μL of nuclei were collected from the 30%/40% gradient interphase and subsequently washed in PBS containing 1% BSA and 1 U μl−1 RNase inhibitor. Following a 5-min incubation on ice, the nuclei were centrifuged at 500g for 3 min at 4°C, and the pellet resuspended in 15 μL of diluted 10X Genomics Nuclei Buffer. Finally, nuclei were counted, and each nuclei suspension was diluted for a targeted nuclei recovery of 10,000 nuclei per sample.

Finally, a subset of nuclei from 3 donors were isolated according to the 10X Genomics’ Chromium Nuclei Isolation Kit Use Guide (PN-1000493, CG000505, Rev. A). Metadata from one donor processed for snRNA-seq was incomplete and was therefore excluded from analyses assessing age-related trends in expression, but was included for cell-type annotation, cell-type deconvolution, and cell-type associated submodule formation (Table S1).

All steps following nuclei isolation were performed the same for each donor tissue processed for snRNA-seq.

#### Tissue processing, imaging, and RNA capture for spatial transcriptomics

Visium spatially resolved gene expression data was generated according to the Visium Spatial Gene Expression User Guide (10x Genomics, CG000239 Rev F). Briefly, tissue sections collected onto Visium Spatial Gene Expression Slides (10x Genomics; 1000185) were fixed in chilled methanol and an abbreviated hematoxylin and eosin protocol was used for histological staining. Brightfield RGB histological images were acquired using an EC Plan-Neofluar 10x/0.3 M27 objective on a Zeiss Axio Observer Z1 fitted with a Zeiss Axiocam 506 mono (Carl Zeiss Microscopy, Germany). Raw CZI images were stitched using Zen 2012 (blue edition) (Carl Zeiss Microscopy, Germany) and exported as JPEGs. Tissue sections were permeabilized for 12 min which was selected as the optimal time based on tissue permeabilization time course experiments conducted using the Visium tissue optimization protocol (10x Genomics, CG000238 Rev E). Reverse transcription was used to generate cDNA molecules incorporating spatial barcodes and unique molecular identifiers (UMIs). After second strand synthesis, cDNA was collected and amplified. The number of cDNA amplification cycles for each Visium array was identified using qPCR as described in the Visium Spatial Gene Expression User Guide.

#### cDNA library preparation

Visium libraries were manually prepared according to the Visium Spatial Gene Expression User Guide (10x Genomics; CG000239 Rev F) using unique Illumina-compatible PCR primers with Dual Index Kit TT Set A, 96 rxn (10x Genomics; 1000215, 10nt index). In addition to qPCR quantification using the KAPA Library Quantification Kit for Illumina Platforms (KAPA Biosystems; KK4824), a Qubit 3.0 fluorometer was used to measure the total yield of the final prepared libraries. Size distribution profiles of the final libraries were assessed using the Agilent 2100 Bioanalyzer system (Agilent High Sensitivity DNA Kit; 5067-4626). Libraries that fell outside of the expected size range (mean library size of 450 bp with a range of 50 bp between libraries) and/or contained adapter dimer contaminations were flagged and excluded from the final pool. Library preparation was repeated for these samples and QCed as described.

#### Sequencing parameters

Size distribution and concentration of single nucleus GEX libraries were assessed on the Fragment Analyzer and quantified using Picogreen and qPCR with the Universal KAPA Library Quantification Kit (KAPA Biosystems; #07960255001). Libraries were pooled, loaded and sequenced on NovaSeq 6000 or NovaSeq X Systems using the Illumina; NovaSeq 6000 S4 Reagent v1.5 Kit (200 Cycles) (Illumina; #20028313); NovaSeq X 25B Reagent Kit (300 Cycles) (Illumina; #20104706). GEX libraries were sequenced using the following recipe: read 1: 28 reads, i7 index read: 10 cycles, i5 index read: 10 cycles, read 2: 90 cycles.

Single nucleus ATAC libraries were sequenced using the following recipe: read 1: 50 reads, i7 index read: 8 cycles, i5 index read: 24 cycles, read 2: 49 cycles. These libraries were successful and the snATAC-seq results passed our initial QC with no problems. As we did not have spatial ATAC-seq results, we determined the snATAC-seq data was less useful to this study, and ultimately decided to exclude them from this paper.

Visium ST libraries were evaluated for size distribution on the Fragment Analyzer and quantified using Picogreen and qPCR with the Universal KAPA Library Quantification Kit (KAPA Biosystems; 07960255001). Libraries were pooled, loaded and sequenced on NovaSeq 6000 or NovaSeq X Systems using the Illumina; NovaSeq 6000 S4 Reagent v1.5 Kit (200 Cycles) (Illumina; #20028313); NovaSeq X 25B Reagent Kit (300 Cycles) (Illumina; #20104706). Libraries were sequenced using the following recipe: read 1: 101 reads, i7 index read: 10 cycles, i5 index read: 10 cycles, read 2: 101 cycles.

#### Telomere-associated DNA damage foci (TAF) staining protocol and analysis

Sections were fixed using 4% paraformaldehyde (PFA) (Electron Microscopy Sciences; #15710) for 1 h at room temperature. Sections were then dehydrated using 5 min incubations of 50%, 70%, then 100% EtOH (Sigma-Aldrich; #E7023). Hybridization buffer was prepared using 20mM Tris-HCl pH 7.4 (Boston BioProducts; #BBT-74), 60% formamide (Sigma-Aldrich; #F7503), 0.1ug/ul salmon sperm DNA (Rockland; #MB-103-0025), and 0.1% tween 20 (Sigma-Aldrich; #P9416) in water. Then 0.25uL of PNA probe TelC-Alexa647 (PNA Bio; #F1013) was added to 25uL of hybridization buffer. The slides and buffer-probe mixture were preheated to 85°C, then 25uL of buffer-probe mixture was added to each slide, adding a coverslip afterward to evenly distribute the buffer. Slides incubated in buffer-probe mix for 10 min at 85°C, then 2 h in the dark at room temperature. Coverslips were removed by incubating slides in 2X SSC buffer (EMD Millipore; #8310). After a series of washes with 2X SSC buffer, sections were stained for *γ*H2AX Ser139 at 1:200 concentration (Cell Signaling Technology; #2577S) and GFAP at 1:1000 concentration (Novus; #NBP1-05198). Imaging of TAF foci was performed on a Leica Stellaris confocal microscope at 63× magnification. For each donor tissue, 10 images were taken in the gray matter, 10 images in the white matter, and 5 images were taken at the border of gray and white matter, which was assessed using the GFAP stain. Images were then analyzed using Cellprofiler 4.2.8, which detected and quantified TelC, *γ*H2AX, and TAF (overlap of TelC and *γ*H2AX) foci within nuclei.

#### Paired somatostatin (SST) *in situ* and immunostaining protocol and analysis

Sections were fixed using 4% paraformaldehyde (PFA) for 1 h at room temperature. Paired *in situ* and immunostaining protocol was adapted from Advanced Cell Diagnostics (ACD). In brief, sections were then dehydrated using 5 min incubations of 50%, 70%, then 100% EtOH. Then, sections incubated in RNAscope hydrogen peroxide (ACD; #322381) for 10 min at room temperature, followed by a 25 min incubation in RNAscope protease plus (ACD; #322381) for 25 min at room temperature. Then, sections incubated in *SST* RNAscope C1 probe (ACD; #310591) for 2 h at 40°C, followed by AMP1 (ACD; #323110) for 30 min at 40°C, then AMP2 (ACD; #323110) for 30 min at 40°C, then AMP3 (ACD; #323110) for 15 min at 40°C, then HRP C1 (ACD; #323110) for 15 min at 40°C, then 1:1500 concentration of 650 TSA dye (ACD; #323273) in TSA buffer (ACD; #322809) for 30 min at 40°C, then HRP blocker (ACD; #323110) for 15 min at 40°C. Sections were then stained for SST at 1:200 concentration (Atlas Antibodies; #HPA019472). Whole tissue scans were performed on a Leica Stellaris confocal microscope at 20× magnification. Images were then analyzed using QuPath 0.5.1, specifically using the positive cell detection tool to identify *SST*+ cells based on nuclear mean fluorescence intensity (MFI) of the *SST* RNAscope probe. From here we quantified the level of *SST* RNA and protein expression per *SST*+ neuron, and the density for *SST*+ cells within each layer of the dlPFC. Table depicting raw quantifications from *in situ* experiments can be found in Table S5. Cortical layer annotations of fluorescently stained tissues were performed by staining an adjacent tissue section from the same tissue block (10 μm apart) for H&E, using the same annotation protocol we used for our Visium sections (see section on Cortical Layer Annotation), then transferring the annotation of the H&E stain to the fluorescence stain.

#### Sequential immunofluorescence (seq-IF) using COMET

The Formalin-Fixed Paraffin-Embedded (FFPE) tissue blocks were obtained from the Edinburgh Sudden Death Brain Bank and sectioned at a thickness of 6 mm. For dewaxing and epitope recovery, an automated PT Module (Epedia; #A80400011) was used. Briefly, tissue slides were incubated for 1 h at 102°C in Dewax and HIER Buffer H (Epredia; #TA-999-DHBH). The slides were removed once the PT Module cooled to 70°C, and then washed twice with multistaining buffer (MSB; Advanced Cell Diagnostics Inc. #BU06) before performing sequential immunofluorescence (seq-IF) staining using an Automated Hyperplex Throughput Immunofluorescence Machine; COMET.^66^ Reagents for automated seq-IF were prepared according to the manufacturer’s instructions. Briefly, antibodies were diluted in multistaining buffer (MSB; Advanced Cell Diagnostics Inc. #BU06); imaging buffer (Advanced Cell Diagnostics Inc.; #BU09) was prepared with Diluent 1 (20X), Diluent 2 (10X) and water; quenching buffer (Advanced Cell Diagnostics Inc.; #BU08-L) was ready with Solution 1 and Solution 2 (10X) in water; Elution buffer (Advanced Cell Diagnostics Inc.; #BU070L) was prepared by combining Buffer 1 and Diluent 2. All the buffers were diluted to a final 1X concentration. Images were taken during each staining cycle. For quality control, autofluorescence images, negative controls (secondary antibodies autofluorescence only), and post-elution images were captured. To control for experimental conditions, Young, Middle, and Old subjects were run simultaneously. During the COMET run, each cycle included three primary antibodies, imaged in the FITC, Cy5, and Cy7 channels. During each cycle of immunofluorescence, the tissue was blocked with 1% BSA (Miltenyi Biotec, # 130-091-376) for 2 min, then incubated with primary antibodies for 4 min, followed by 1 min of blocking, and finally with secondary antibodies for 1 min. After every cycle, the antibody was eluted using the elution buffer and imaged for post-elution quality control. For CNS cell markers, Map2 (1:5000), GFAP (1:10000), IBA1 (1:250), Olig2 (1:50), and CD31 (1:250) were used for neurons, astrocytes, microglia, oligodendrocytes, and endothelial cells, respectively. Secondary antibodies: Donkey anti-Rabbit IgG (Thermo Fisher Scientific, #A31573), anti-Mouse IgG (Thermo Fisher Scientific, #A10037), anti-Chicken IgY (Thermo Fisher Scientific, #A78948), and anti-Goat IgG (Thermo Fisher Scientific #A-11055) were used at a 1:1000 dilution. Images were captured at 20X resolution. All images were automatically processed and aligned using the COMET software (Lunaphore Technologies), and the output was generated as a single.ometiff file for further analysis.

For visualization and subtraction of autofluorescence and negative controls, HORIZON Viewer (Lunaphore Technologies) software was used. The images were analyzed for quantitative outcomes using HALO software (Indica Labs; #PR02122) with artificial intelligence (AI) add-ons. For nuclei and membrane segmentation, the software was trained based on the size, shape, and intensity of DAPI and membrane markers across all cell types. For FN1 measurements, thresholds were set using the software’s “Threshold Calculation’ function for all images. The intensity of all stained markers across all cell types was analyzed across all donors, and the DAPI intensity for each cell type and donor were normalized to obtain final SPP1, p16, and BCL2 intensities. The results were generated as.csv files. Table S11 displays raw quantifications of each protein target’s MFI within each profiled cell. For visualization, the.csv files were processed in RStudio.

### Quantification and statistical analysis

#### Power analysis

To justify the sample size that we used for our Visium ST experiments, we performed a bayesian power analysis through simulations for our bayesian statistical model, splotch (see section titled “Splotch modeling of spatial gene expression data”). We fit splotch using simulated spatial gene count data, and determined power to correctly infer expression difference between a control and comparison group at our threshold (BF > 3) (Figure S11A).

In order to determine the biologically relevant range of spatial expression for simulation, we calculated maximum likelihood estimates of the zero-inflated poisson parameters lambda and theta for all genes separately for each annotated anatomical region, from a real-world spatial prefrontal cortex dataset (Figure S11B). We selected three representative expression levels, labeled LOW (mean expression of 0.025 counts/spot; 20th percentile of expression), MEDIUM (mean expression of 0.6 counts/spot; 90th percentile of expression) and HIGH (mean expression of 10 counts/spot; 99.9th percentile of expression) for simulation, and simulated fold changes from 1 to 3 between control and comparison.

As expected, a low number of spatial arrays per group is required to confidently assess changes in gene expression in simulated “HIGH” and “MEDIUM” genes (Figures S11A and S11B). For instance, models fit with 20 spatial arrays per age group have 80% power across all cortical layers to detect fold changes of 1.2 or greater (subfigure A). “LOW” expressed genes require more spatial arrays per group to achieve 80% power (Figure S11A). We determined that at our current sample size (37 young arrays, 62 middle arrays, 73 old arrays) we were powered to detect log2FC differences of ±1 at a BF threshold of 3 for greater than 80% of the genes in our dataset.

#### Quality check of single-nucleus transcriptomic data

Barcoded processing, gene counting and aggregation were made using the Cell Ranger software v2.0.0. Cell Ranger output was then processed through CellBender 0.3.0 to eliminate technical noise. All the 10X runs for each human sample were initially filtered with an nUMI cutoff of >500 and then nuclei with less than 5% mitochondrial (MT) gene contamination were retained. Next, the mitochondrial genes were also removed from the count matrices. DoubletFinder v3 was initially used to identify doublets; we leveraged these classifications during the clustering step to remove clusters with a high proportion of doublets and then any additional remaining doublets. Data was processed through the Seurat SCT workflow, including normalizing data, identifying variable features, and scaling. This was followed by running PCA, finding clusters, and projecting data onto UMAP space. Nuclei were manually annotated into seven broad classes: excitatory neurons, inhibitory neurons, oligodendrocytes, oligodendrocyte precursor cells (OPCs), astrocytes, microglia, vascular cells. We used marker genes: SNAP25 (for neurons), SLC17A7 (for excitatory neurons), GAD1 and GAD2 (for inhibitory neurons), MOG and MAG (for oligodendrocytes), PDGFRA (for OPCs), AQP4 and FGFRR3 (for astrocytes), AIF1 (for microglia), CLDN5 (for endothelial cells), and PDGFRB (for pericytes). After identifying broad classes, we split the data into these eight classes and re-ran the SCT workflow to identify further contaminations in the data. A total of 117,914 nuclei passed quality control filtering, with average detection of 4,667 UMIs per nucleus and 3,186 nuclei per sample (Table S1).

#### Mapped annotations of single-nucleus transcriptomic data

For further cell subtype annotation, we mapped our nuclei to the aged prefrontal cortex dataset released by Green et al. (2024).^55^ This comprehensive dataset includes 1.64 million single-nucleus RNA-seq profiles from 424 aging individuals. We used the Allen Institute cell type mapping algorithm, MapMyCells, (RRID: SCR_024672), a fast web-based tool. In addition, we ran this algorithm five times on our entire dataset and then used majority-vote from the five runs to select a final consensus annotation.

The output from the mapping algorithm annotated several nuclei into small subtype populations, as well as cell types that are not widely expected in the cortex. For small clusters not pertaining to any of the major broad classes, these were labeled as “Other”. Number of nuclei annotated for each of the resulting 81 mapped clusters and 8 broad class cell types reported in Table S3.

#### Single-nucleus differential expression analysis

DE analysis for the single-nucleus RNA sequencing data was performed with the voom function from the limma package (ISSN: 1474-760X). Data was filtered, normalized, and aggregated using the edgeR package (PMCID: PMC2796818). We filtered for genes that were expressed in at least 30 nuclei and present in at 75% of either the young or old samples. Afterward, we performed linear modeling with limma where age is treated as a continuous variable. The voom function incorporates the mean-variance relationship in RNA-seq count data by assigning precision weights in order to assume normally distributed log-CPM values. All models included an intercept term along with a sex control variable and a batch fixed effect. P-values were corrected for multiple testing using the Benjamini-Hochberg method. A summary of aging markers by cell type at the broad class and mapped subcluster level can be found in Table S7.

#### Gene ontology analysis of single nucleus age-related DEGs

GO BP enrichment analysis of differentially expressed genes downregulated with age (adj p-val<0.05, Log2FC < −1) was conducted using Metascape v3.5.20250701. Such downregulated DEGs from each broad class cell type (as well as the list of shared differentially expressed genes across 2 or more broad class cell types) were used as “input list” while all other genes in our snRNA-seq dataset were used as “background”. Enrichment was performed using the custom analysis tool, solely exploring enriched pathways from the “GO Biological Process” database, and with a *p*p-value cutoff of 0.01. A summary of GO BP terms enriched within each broad class and shared gene program can be found in Table S8.

#### Mixed association enrichment analysis

For associations between two categorical variables, we used MASC, a tool for mixed effects modeling of associations of single cells. For the analysis looking at enrichment of cell populations between young and the older age groups, we used sex and sample & batch as the fixed and random effects, respectively. For further downstream analysis looking at enrichment of senescence cell populations, we used sex as a fixed effect when not sex-stratified, and using batch as the random effect.

#### Single-nucleus gene module scoring of senescence hallmarks

Module scores were computed using Seurat’s *AddModuleScores* function. Scores are calculated by subtracting the average expression levels of gene sets at the single cell level with the average expression of a control feature set. This calculation is specifically done by, first, binning all genes (gene set and control set) based on average expression and then finding differences between the query genes and randomly selected control features. For calculating module scores for the snRNA-seq data of the 10 annotated gene lists, we maintain the same 2,000 control feature set when running *AddModule Scores*.

The goal of our analysis was to identify positive for senescence hallmarks in the aged dlPFC. We utilized 3 multi-hallmark senescence gene lists curated from the literature: SenMayo, Fridman Senescence Up, and a gene list curated by SD TMC. Additionally, we adapted 6 of the 9 individual hallmark gene lists from the recent SenNet review paper: Cell-Cycle Arrest, SASP, Resistance to Apoptosis, Damage DNA Response, Cell Surface Markers, and Increased Lysosomal Content. Since the idea of this analysis was that nuclei scoring high for these hallmark gene lists were “positive” for a particular hallmark, we chose to exclude gene lists for nuclear changes, metabolic adaptations, and changes in cell morphology, for which we believed changes in mRNA were insufficient to assess. Finally, a list of Activated P53 Targets was also used as an individual hallmark gene list. Each of these gene lists can be found in Table S9.

Across each broad class and senescence hallmark, we determine the classification of nucleus as senescent by establishing a threshold based on the top 5% percentile from the module scores of the young age group. For each hallmark, we classify a nucleus as “positive” for that specific senescence hallmark if its module score is higher than the hallmark’s threshold (i.e., a threshold for each hallmark). For each hallmark and broad cell type, we test for differences in proportions of “positive” nuclei between samples from the young and middle/old age group using MASC. We also identify nuclei that are positive for at least three individual senescent hallmarks of the seven, which we refer to as “3+ Hallmarks”.

#### Identifying DEGs characteristic of p16+ oligodendrocytes

For p16 positivity status in oligodendrocytes, we label nuclei as “p16+” if the CDKN2A transcript count is greater than zero, “p16-“ otherwise. For DEGs based on p16 positivity status, we use Seurat’s *FindAllMarkers* using the ROC test. Standard linear regressions were performed regressing a donor’s percentage of “p16+” nuclei against age of death. DEGs displayed in Table S10.

#### Spatial sequence data preprocessing

Raw FASTQ files and histological images were processed using Space Ranger v.2.0.0, which uses a modified STAR v2.7.2a for genome alignment and performs barcode/UMI counting to generate feature-spot matrices. Reads were aligned to the GRCh38 2020-A human reference genome pre-built by 10x Genomics. Read alignment yielded an average of 1225 median genes and 2545 median UMIs per ST spot across our dataset of 705,943 ST spots prior to filtering of genes and spots for Splotch analysis (Table S1).

Tissue arrays demonstrating extensive RNA degradation (with median genes detected per spot <700) were excluded from our analysis. Tissue sections with freeze fracture or other tissue sectioning artifacts that precluded cortical layer annotation of a majority of spots were also excluded from analysis. Where possible, these dropped replicates were replaced with additional technical replicates processed and sequenced on a later date. After array level QC, our dataset consists of 172 tissue sections from 11 young (<45), 19 middle (45–55), and 21 old (>55) donors.

#### Cortical layer annotation

Following sequencing, each tissue section was manually annotated to capture transcriptomic changes occurring within unique cortical anatomical layers. All Visium spots under tissue sections were annotated with one of 7 anatomical annotation regions (AARs) consisting of the 6 cortical layers and the white matter. Annotation was performed using Loupe Browser (10x Genomics, Version 6.3.0) which enables interactive visualization of H&E images acquired as part of the Visium workflow with corresponding spot level gene expression data as registered by Space Ranger. Cortical layer annotations were assigned on the basis of cellular morphology in the portion of the histological image associated with each Visium spot. Cortical layers were identified as follows. The white matter was identified based on the lack of neuronal cell bodies and the linear arrangement of glial nuclei along neuronal processes. Cortical layer 6 was defined as the heterogeneous neuronal layer spanning from the white matter to the beginning of cortical layer 5, marked by large pyramidal neurons oriented perpendicularly to the pial surface. Cortical layer 4 was identified based on the presence of small, granular neurons adjacent to the large pyramidal neurons of cortical layer 5. Cortical layer 3 was labeled as the pyramidal cell layer between granular layers 2 and 4. Finally cortical layer 1 was identified based on the paucity of cells and lack of large neuronal soma. Spots that contained meninges were labeled as such and excluded from downstream analysis due to the distinct cellular composition of meninges as compared to cortical layer 1. Spots overlying tissue artifacts such as freeze fracture, folds, or tears were excluded from the analysis.

#### Spatial data integration and UMAP embedding

Visium ST data were integrated and projected into UMAP space using the anchor-based workflow of Seurat v4.4.0. Expression data were normalized and variable features selected for each Visium array using the NormalizeData and FindVariableFeatures methods, respectively. Integration features were selected across all arrays using SelectIntegrationFeatures, then scaling and PCA were performed over said features using ScaleData and RunPCA applied separately to each array. Due to the size of the dataset, reciprocal PCA (RPCA) with reference-based dataset anchors (FindIntegrationAnchors with 50 dimensions) was applied using all four arrays from one 50-year-old male control donor (U54-HRA-010) as a reference. Finally, Visium data were integrated using IntegrateData, scaling was applied across the integrated dataset, and PCA was applied using default parameters, followed by further UMAP reduction of the first 50 PCs using min_dist = 0.5 and n_neighbors = 50 and all other parameters set to default values.

#### Splotch modeling of spatial gene expression data

Raw sequencing data was filtered to remove mitochondrially encoded genes, lncRNAs, pseudogenes, and genes expressed in less than 0.67% of spots (expression level of senescence-associated CDKN2A). A summary of spot expression percentages for each gene in our dataset can be found in Table S2. Spots with fewer than 100 UMIs across remaining genes were discarded prior to Splotch analysis. Finally, spots without a valid annotation were discarded, and any spots without at least one immediate neighbor in the Visium grid were discarded to prevent singularities in the spatial autocorrelation component of the Splotch model. This resulted in expression data for 13,310 genes measured at 671,397 spatial spots across all patients with an average sequencing depth of 2,754 UMIs per spot.

Splotch employs a zero-inflated Poisson likelihood function (where the zero-inflation accounts for technical dropouts) to model the expression (***λ***) of each gene in each spot. This expression level is in turn modeled using a generalized linear model (GLM) with the following three components: the characteristic expression of the spot’s AAR (β), autocorrelation with the spot’s spatial neighbors (*Ψ*), and spot-specific variation (ε). Furthermore, the characteristic expression rate (β) is hierarchically formulated to account for the experimental design, enabling the investigation of the effect of sample covariates on differential gene expression. By performing posterior inference on the model given the observed spatial expression data, we identified clinical group level trends in spatial gene expression (β) that best explains our observations. (β) is measured in log-scaled counts of gene expression per spot within an AAR of interest.

Compared to other computational methods for analysis of spatial transcriptomics data, Splotch i) enables quantification of expression differences between conditions and anatomical regions, ii) is designed to rigorously account for the uncertainty of low counts, and iii) analyzes multiple tissue sections simultaneously, stratifying patients based on clinical phenotype in order to quantify biological and technical variation.

#### Spatial differential expression analysis

Differential expression analysis between conditions was performed by quantifying differences in the estimated posterior distributions (β) for young, middle and old donors. This was accomplished using the Savage-Dickey density ratio to calculate a Bayes factor (BF) for a given comparison^43^ (e.g., expression of *SST* in cortical layer 2, Old vs. Young). A BF > 3 was the threshold used to identify differentially expressed genes between conditions. Bayes factor tables summarizing differentially expressed genes with age can be found in Table S4.

#### Spatiotemporal co-expression analysis

To study spatiotemporal and disease-dependent co-expression patterns in the human cortex, we consider all the mean posterior spot-level expression estimates (***λ***) from our Splotch model – a matrix with 671,397 rows (spots) and 13,310 columns (genes). The gene-gene Pearson correlation matrix was calculated and KNN clustering was performed with k = 10 neighbors to group genes of similar co-expression pattern across spots. Module membership was then determined by Leiden clustering with a resolution of 2. Any module with <50 genes was discarded from further analysis. A summary of gene placement within our spatial coexpression modules can be found in Table S12.

For each coexpression module, cell type-associated submodules were derived using our donor-matched snRNA-seq dataset of 117,914 nuclei. Raw expression data were depth-normalized to 10k counts per nuclei then log1p-transformed. Separately for each spatial coexpression module, Pearson correlations between member genes were calculated using the transformed snRNA-seq data, then KNN clustering was performed with k = 10 neighbors to group genes with similar co-expression patterns across nuclei. Submodule membership was then determined by Leiden clustering with a resolution of 1. A summary of gene placement within our submodules can be found in Table S12.

Significance testing for differential module/submodule expression across age groups was accomplished by first calculating per-spot module scores, then averaging per-array and per-cortical layer to produce independent batch estimates and applying Welch’s *t* test (with Benjamini-Hochberg FDR correction) between age groups (e.g., “Young” vs. “Old” in Layer_1). Per-spot module scores were calculated by first clipping (99th percentile) and standard-scaling the expression of each module/submodule gene across all spots to remove the effects of variation in basal gene expression, then averaging the expression of member genes within each spot.

#### Gene ontology analysis of spatial modules

GO BP enrichment analysis of coexpression modules and cell type specific submodules was conducted using Metascape v3.5.20250701. Genes from modules or submodules used as “input list” while all other genes in our ST dataset were used as “background”. Enrichment was performed using the custom analysis tool, solely exploring enriched pathways from the “GO Biological Process” database, and with a *p*p-value cutoff of 0.01. For the purpose of this analysis, only coexpression modules sized between 100 and 3000 genes were considered. A summary of GO BP terms enriched within each spatial coexpression module can be found in Table S13.

#### Cell type deconvolution of Visium data

Per-spot estimates of the abundances of k = 8 broad and k = 32 merged fine-grained cellular subtypes (Table S3) were derived using Cell2location. Cell2location accepts two data matrices: a (spots, genes) matrix of ST data and a (cells, genes) matrix of sc/snRNA-seq data. These data matrices were first subset to the set of genes detected in both modalities, then further subset to a set of marker genes “balanced” across the broad cell types (i.e., the same number of markers for each cell type) to enhance the differences between cellular subtypes.

Marker genes for deconvolution were derived from snRNA-seq data by first pseudo-bulking per-cell type and per-donor (dropping any cell types without at least 10 cells in at least two donors), then depth-normalizing to 1e4 counts per pseudo-sample, log-transforming, and applying scanpy’s rank_genes_groups method. Per-cell type, genes with >1.25 mean log-fold change (vs. other cell types) and p_adj<0.05 were retained, then each gene was assigned a dispersion score (variance-mean ratio within each cell type, averaged across cell types), and the top 1% of genes by dispersion were discarded following the methodology of Ma et al. (2018). Marker genes for (k = 8) broad classes and (k = 32) fine-grained classes can be found in (Table S6).

Cell2location’s negative binomial (NB) regression model was trained on our full counts matrix (all donors) for 250 epochs with a batch size of 2,500 to produce reference signatures for all cell types. Deconvolution of spatial data was performed per-donor with the aforementioned reference, treating individual Visium arrays as separate batches. The mean cells per spot was set to five, detection alpha set to 20 (default), and the model was trained for 10,000 epochs.

Significance testing for differential cell type abundance across age groups was accomplished by first normalizing spot composition vectors across cell types to sum to 1, then averaging per-donor and per-cortical layer to produce independent batch estimates and applying the Wilcoxon rank sums test (with Benjamini-Hochberg FDR correction) between age groups (e.g., “Young” vs. “Old” in Layer_1).

#### TANGRAM deconvolution of spatial data

In order to better leverage the paired nature of the snRNA-seq and ST data, we performed an additional deconvolution analysis with TANGRAM, a method for imputing the location of individual cells/nuclei within intact ST-profiled tissues. Unlike Cell2location, which leverages the full snRNA-seq dataset to construct a robust reference for imputing cell abundance, TANGRAM utilizes only those nuclei from adjacent tissue sections, reducing sample size but removing sources of biological variation. Due to the nature of the method, we limited ourselves to the *N* = 36 donors from which both ST and snRNA-seq data were available and performed imputation separately for data from each such donor. TANGRAM was limited to using marker genes from Cell2location (k = 8 broad classes; Table S6) and was otherwise run with default parameters. Identically to Cell2location, significance testing for differential cell type abundance across age groups was accomplished by first normalizing spot composition vectors across cell types to sum to 1, then averaging per-donor and per-cortical layer to produce independent batch estimates and applying the Wilcoxon rank sums test (with Benjamini-Hochberg FDR correction) between age groups (e.g., “Young” vs. “Old” in Layer_1). Results displayed in Figure S4B.

#### Gene set spatial enrichment analysis

Per-spot module scores were calculated for each of 10 senescence-related gene sets, 2 senescence-related spatial modules, and 3 senescence related spatial submodules (Table S12) using Scanpy’s *score_genes* function. For the control feature set, we randomly selected 2,000 genes not included in any of the senescence gene sets.

Deciles were computed for each module across all *n* = 671,397 spots in the dataset, and each spot was assigned a discrete label between 1 (d1) and 10 (d10) for each module based on the decile of the observed expression score. Separately for each age group and module, spatial enrichment scores for all pairs of deciles were calculated using Squidpy’s^107^ nhood_enrich function with *n* = 1,000 permutations, treating individual Visium arrays as batches.

## Supplemental information

Document S1. Figures S1–S11

Table S1. Donor metadata and QC metrics, related to Figure 1 In-depth subject metadata sheet with basic ST/snRNA-seq subject by subject QC metrics.

Table S2. Spot-level expression percentages, related to Figures 1 and 2 ST spot expression percentage of each gene in our Splotch run.

Table S3. Nuclei per broad class and subcluster label, related to Figures 1, 3, and 4 Number of nuclei in our dataset annotated as dlPFC broad class cell types and Green et al.^55^ subclusters. Cluster merging scheme for cell2location analysis in Figure 3.

Table S4. ST DEG analysis, related to Figure 2 Splotch differential expression tables for different age and sex conditions.

Table S5. *SST in situ* quantification, related to Figure 2 Raw data for *SST in situ* experiments with mean fluorescence intensity (MFI) and cell-density measurements by cortical layers.

Table S6. Cell2location markers, related to Figure 3 Marker genes used for cell2location deconvolution of merged mapped cluster cell types

Table S7. snRNA-seq DEG analysis, related to Figure 4 snRNA-seq DEG analysis at the broad class level and subcluster level.

Table S8. GO BP pathways enriched in broad class cell types, related to Figure 4 GO biological process (BP) pathways enriched within genes significantly decreasing with age (adjusted *p* <0.05, log2FC < −1) among individual snRNA-seq broad class cell types and among shared genes decreasing with age in Figure 4A.

Table S9. Senescence hallmark gene lists, related to Figures 4, 5, and 6 Table summarizing all curated senescence gene sets used in our analysis.

Table S10. DEGs of CDKN2A-positive oligodendrocytes, related to Figure 4 Table listing differentially expressed genes between CDKN2A(p16)+ and − oligodendrocytes.

Table S11. COMET quantification, related to Figure 4 Raw comet data for each result displayed in the paper separated by cell type.

Table S12. Module and submodule gene lists, related to Figure 5 Gene placement in spatial coexpression modules and cell-type-specific submodules.

Table S13. GO BP pathways enriched in spatial modules, related to Figure 5 GO BP pathways enriched within each spatial module.

Document S2. Article plus supplemental information
