# Supplementary material for: Uncovering the signatures of aging and senescence in the human dorsolateral prefrontal cortex
Source: Cell Genom. 2026 Jan 22;6(2):101127. doi: 10.1016/j.xgen.2025.101127 (PMC12903411; doi:10.1016/j.xgen.2025.101127)
Supplement: Document S1. Figures S1–S11 [file mmc1.pdf]

Cell Genomics, Volume *6*

Supplemental information

Uncovering the signatures of aging and senescence in the human dorsolateral prefrontal cortex

Nicholas X. Sloan, Jason Mares, Aidan C. Daly, Shaunice Grier, Imdadul Haq, Christopher A. Jackson, Natalie Barretto, Obadele Casel, Kristy Kang, Shruti Khiste, Kennedy Harris, Jacqueline Eschbach, Benjamin T. Fullerton, Courteney Mattison, Brhan Gebremedhin, Joana Petrescu, Lilian Coie, Maria Hauge Pedersen, Ke Zhang, Jian Shu, Andrew F. Teich, Hasini Reddy, Colin P. Smith, Yousin Suh, Vilas Menon, and Hemali Phatnani

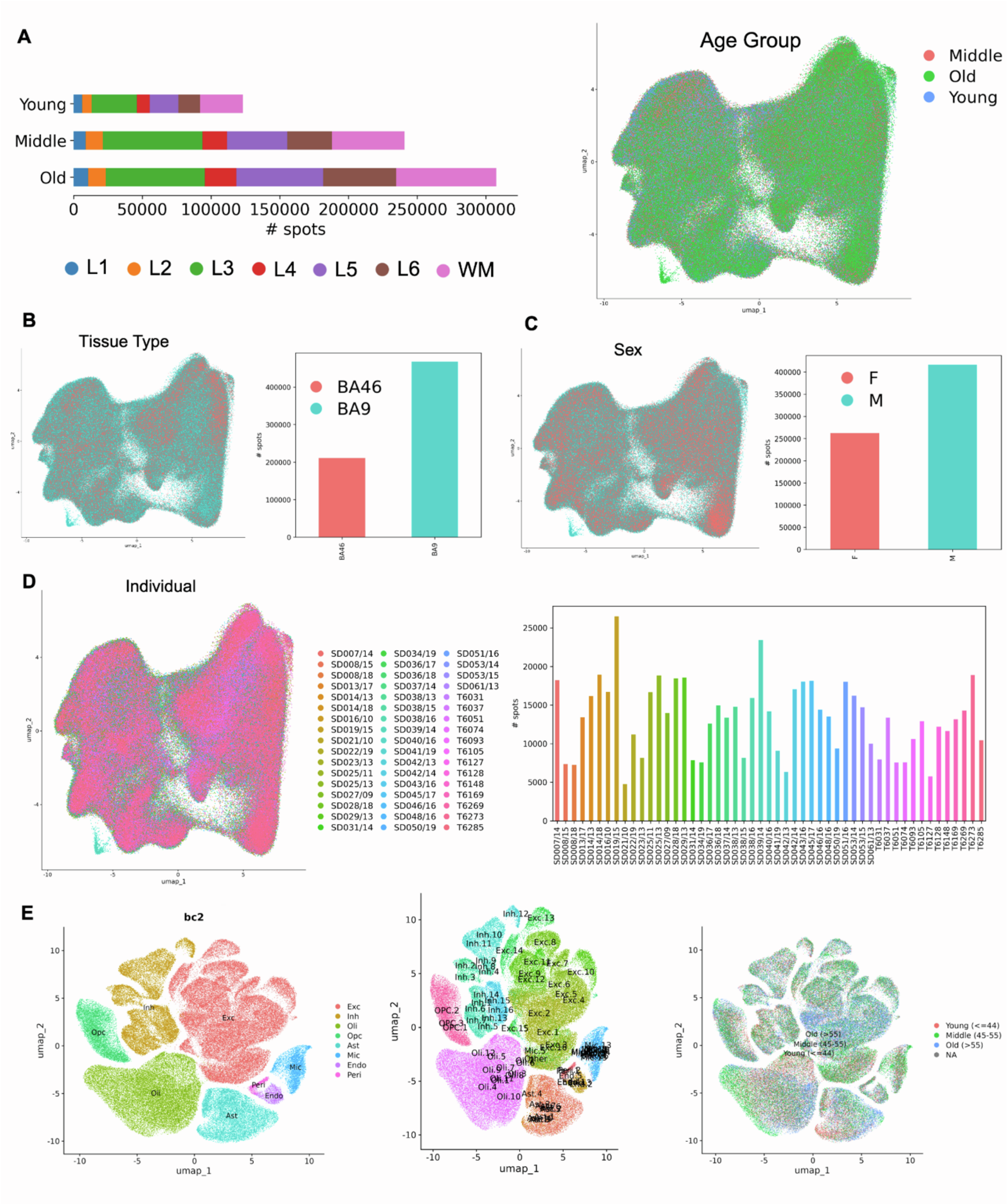

Figure S1 Additional QC of ST and snRNA-seq datasets, related to Figure 1
(**A**) (Left) Bar plot depicting number of annotated ST spots from each age group corresponding to each AAR (see legend). (Right) UMAP plot of Visium data colored by age group.
(B-D) Remaining UMAP plots of Visium data and corresponding bar plots quantifying number of ST spots per variable are colored by (**B**) tissue type, (**C**) sex, and (**D**) subject, respectively.
(**E**) From left to right, UMAP plots of snRNA-seq data colored by broad class, the mapped cluster label from the Green et al. (2024) paper, and age group, respectively.

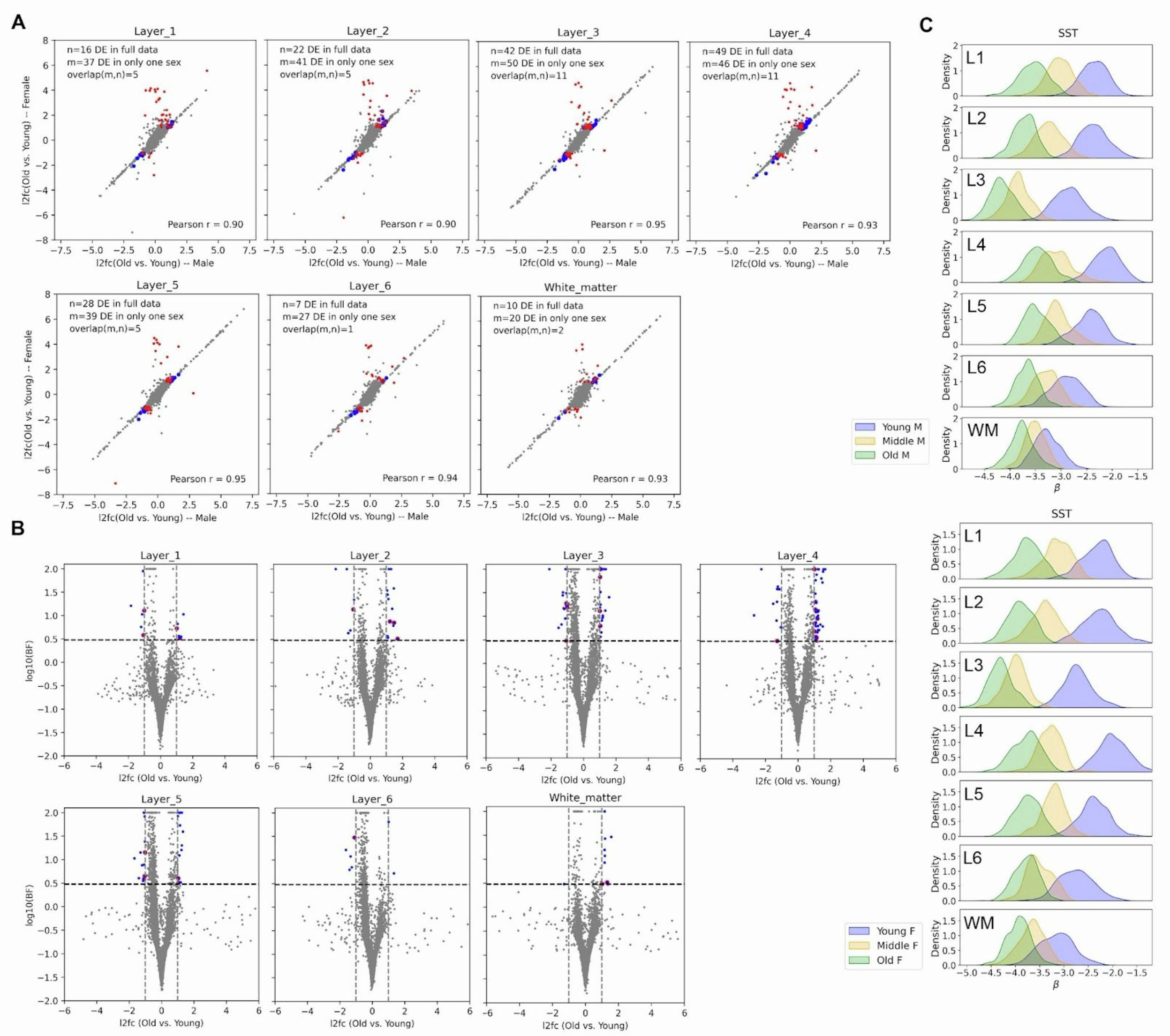

Figure S2 Effect of sex on temporal differential expression patterns, related to Figure 2

(A) Correlation between inferred magnitude of change (l2fc: log2 fold change) between “Old” and “Young” groups in male (x-axis) and female (y-axis) patient groups. Blue dots indicate genes exhibiting significant temporal DE when considering full data (male and female), red dots denote genes exhibiting significant temporal DE exclusively in one sex. Results are displayed separately for each cortical layer (subplots).

(B) Volcano plots displaying magnitude (x-axis) and significance (y-axis) of temporal expression change for all genes in (A) when considering full data. Color code is shared with (A). Dashed lines (vertical-gray and horizontal-black) denote magnitude (l2fc) and significance (BF: Bayes factor) cutoffs for determining temporal DE.

(C) Inferred distributions over expression of SST in each age group (color code), stratified by sex (upper: male, lower: female) and cortical layer (subplots L1-L6, WM)

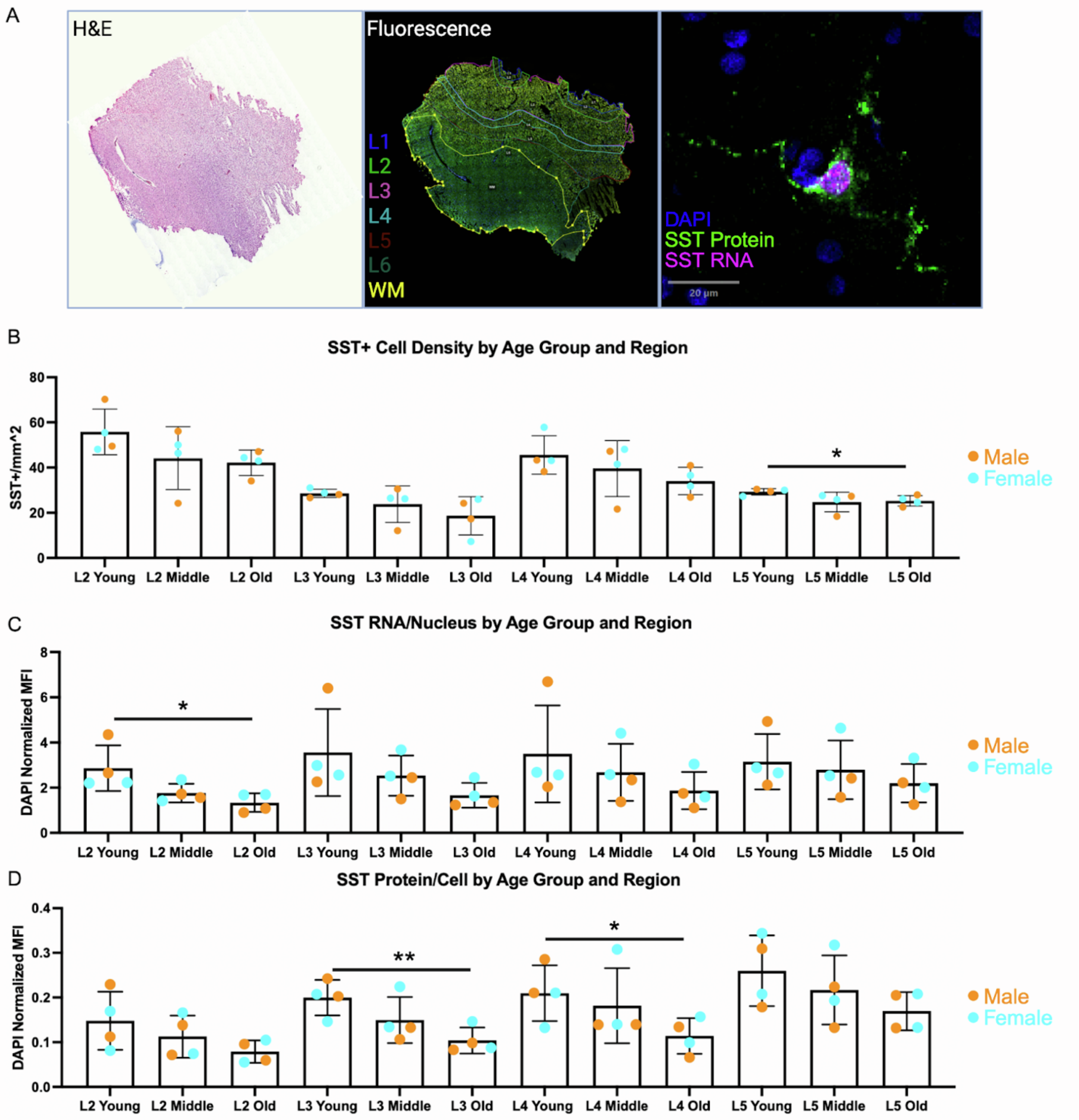

Figure S3 Loss of *SST*+ cell type density and loss of *SST* RNA/protein per *SST*+ cell in observed in aged grey matter layers 2-5, related to Figure 2

(**A**) Fluorescence tissue sections (middle) are annotated by eye using adjacent H&E-stained sections (left). Tissues were stained for an *SST* RNAscope probe and SST antibody (right).

(**B**) Quantification of *SST*+ cell density (determined using *in situ* probe) in layers 2-5 of the cortex,

(**C**) Quantification of *SST* RNA level per nucleus within *SST*+ cells in layers 2-5 of the cortex, and

(**D**) Quantification of SST protein level per cell within *SST*+ cells in layers 2-5 of the cortex, across young (n=4), middle (n=4), and old (n=4) subjects. Error bars represent standard deviation, and dots represent individual measurements for each subject, colored by sex (see legend). Significance determined using unpaired 2-sided t test with Welch's correction. * = p-val<0.05, ** = p-val<0.1.

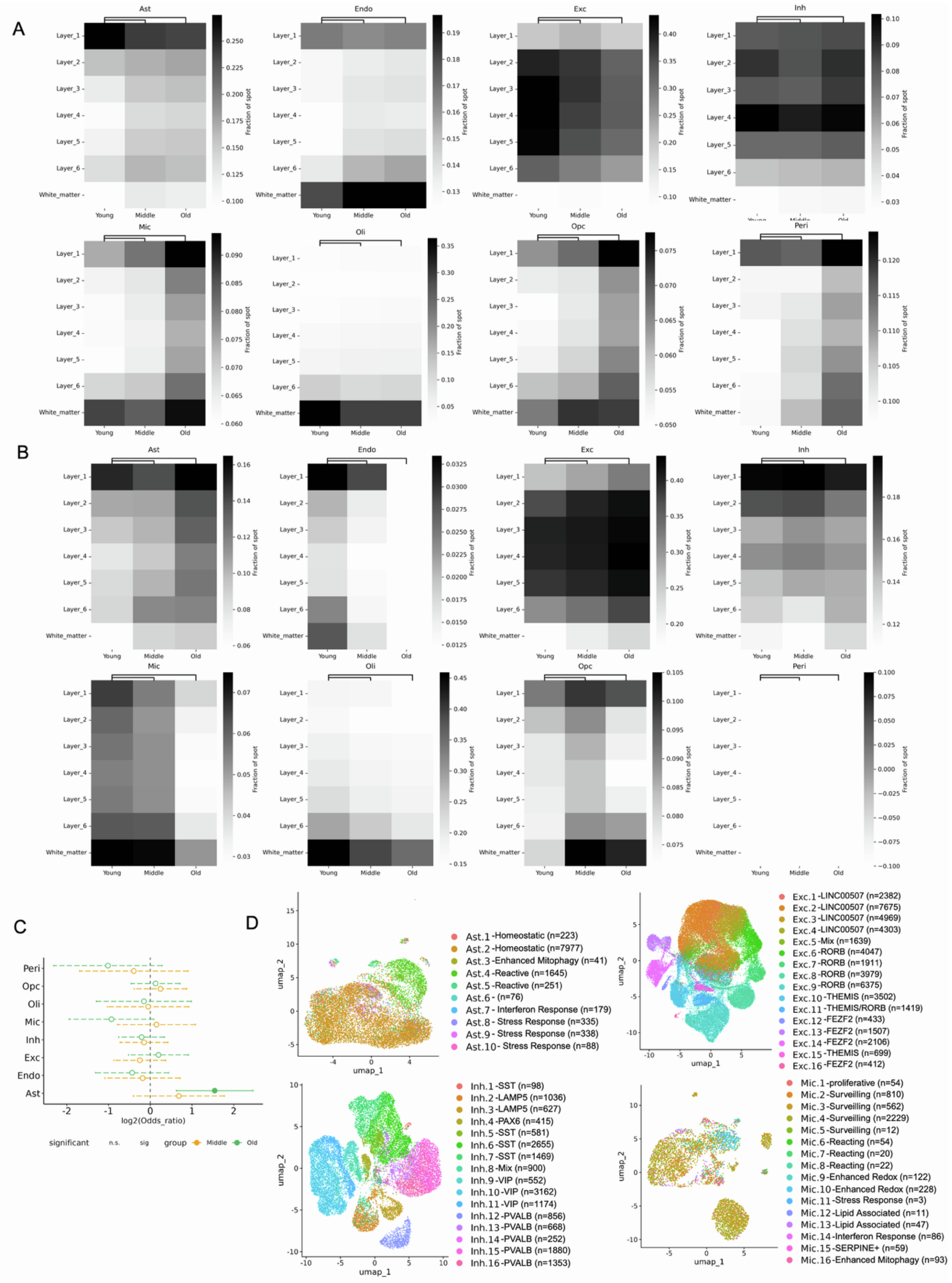
A
Ast
Endo
Exc
Inh
Mic
Oli
Opc
Peri
Layer_1
Layer_2
Layer_3
Layer_4
Layer_5
Layer_6
White_matter
Young
Middle
Old
Fraction of spot
B
C
Peri
Opc
Oli
Mic
Inh
Exc
Endo
Ast
log2(Odds_ratio)
significant
n.s.
sig
group
Middle
Old
D
umap_1
umap_2
Ast.1 -Homeostatic (n=223)
Ast.2 -Homeostatic (n=7977)
Ast.3 -Enhanced Mitophagy (n=41)
Ast.4 -Reactive (n=1645)
Ast.5 -Reactive (n=251)
Ast.6 - (n=76)
Ast.7 - Interferon Response (n=179)
Ast.8 - Stress Response (n=335)
Ast.9 - Stress Response (n=338)
Ast.10 - Stress Response (n=88)
Exc.1 -LINC00507 (n=2382)
Exc.2 -LINC00507 (n=7675)
Exc.3 -LINC00507 (n=4969)
Exc.4 -LINC00507 (n=4303)
Exc.5 -Mix (n=1639)
Exc.6 -RORB (n=4047)
Exc.7 -RORB (n=1911)
Exc.8 -RORB (n=3979)
Exc.9 -RORB (n=6375)
Exc.10 -THEMIS (n=3502)
Exc.11 -THEMIS/RORB (n=1419)
Exc.12 -FEZF2 (n=433)
Exc.13 -FEZF2 (n=1507)
Exc.14 -FEZF2 (n=2106)
Exc.15 -THEMIS (n=699)
Exc.16 -FEZF2 (n=412)
Inh.1 -SST (n=98)
Inh.2 -LAMP5 (n=1036)
Inh.3 -LAMP5 (n=627)
Inh.4 -PAX6 (n=415)
Inh.5 -SST (n=581)
Inh.6 -SST (n=2655)
Inh.7 -SST (n=1469)
Inh.8 -Mix (n=900)
Inh.9 -VIP (n=552)
Inh.10 -VIP (n=3162)
Inh.11 -VIP (n=1174)
Inh.12 -PVALB (n=856)
Inh.13 -PVALB (n=668)
Inh.14 -PVALB (n=252)
Inh.15 -PVALB (n=1880)
Inh.16 -PVALB (n=1353)
Mic.1 -proliferative (n=54)
Mic.2 -Surveilling (n=810)
Mic.3 -Surveilling (n=562)
Mic.4 -Surveilling (n=2229)
Mic.5 -Surveilling (n=12)
Mic.6 -Reacting (n=54)
Mic.7 -Reacting (n=20)
Mic.8 -Reacting (n=22)
Mic.9 -Enhanced Redox (n=122)
Mic.10 -Enhanced Redox (n=228)
Mic.11 -Stress Response (n=3)
Mic.12 -Lipid Associated (n=11)
Mic.13 -Lipid Associated (n=47)
Mic.14 -Interferon Response (n=86)
Mic.15 -SERPINE+ (n=59)
Mic.16 -Enhanced Mitophagy (n=93)

Figure S4: Changes in broad class cell type composition and mapped cluster functional annotations, related to Figure 3 and STAR Methods

(A-B) Heatmaps visualize abundance of all broad class cell types predicted by **(A)** cell2location or **(B)** TANGRAM (STAR Methods) using ST data across cortical layers (y-axes) and age groups (x-axes). Significant variation (2-sided Welch's t-test, BH FDR correction) in cell composition between donors in the Middle/Old and Young age groups is denoted with star (*) notation (*:p_adj < 0.05). TANGRAM results generally agreed with cell2location in terms of the spatial and temporal distribution of cell types, and neither method predicted significant compositional changes across age groups. This is not sufficient to fully discount biological variation in cell type proportion as a contributor towards observed differential gene expression – indeed, these methods are inherently limited by technical differences between snRNA-seq and ST capture technologies – but does provide important validation of the generality of our cell typing and marker gene selection.

(C) MASC differential cell type proportion testing results between young and middle (yellow) and young and old (green) groups are shown for broad class cell types. Points to the left (right) of the dotted line indicate decreased (increased) cell type enrichment, respectively, in the older group. Significant results (p<=.05) are indicated by solid, filled-in bubbles and solid confidence interval bars.

(D) UMAP plots of astrocytes, excitatory neurons, inhibitory neurons, and microglia colored by mapped cluster annotation derived from Green at al. (2024). Number of annotated nuclei per subcluster in parentheses.

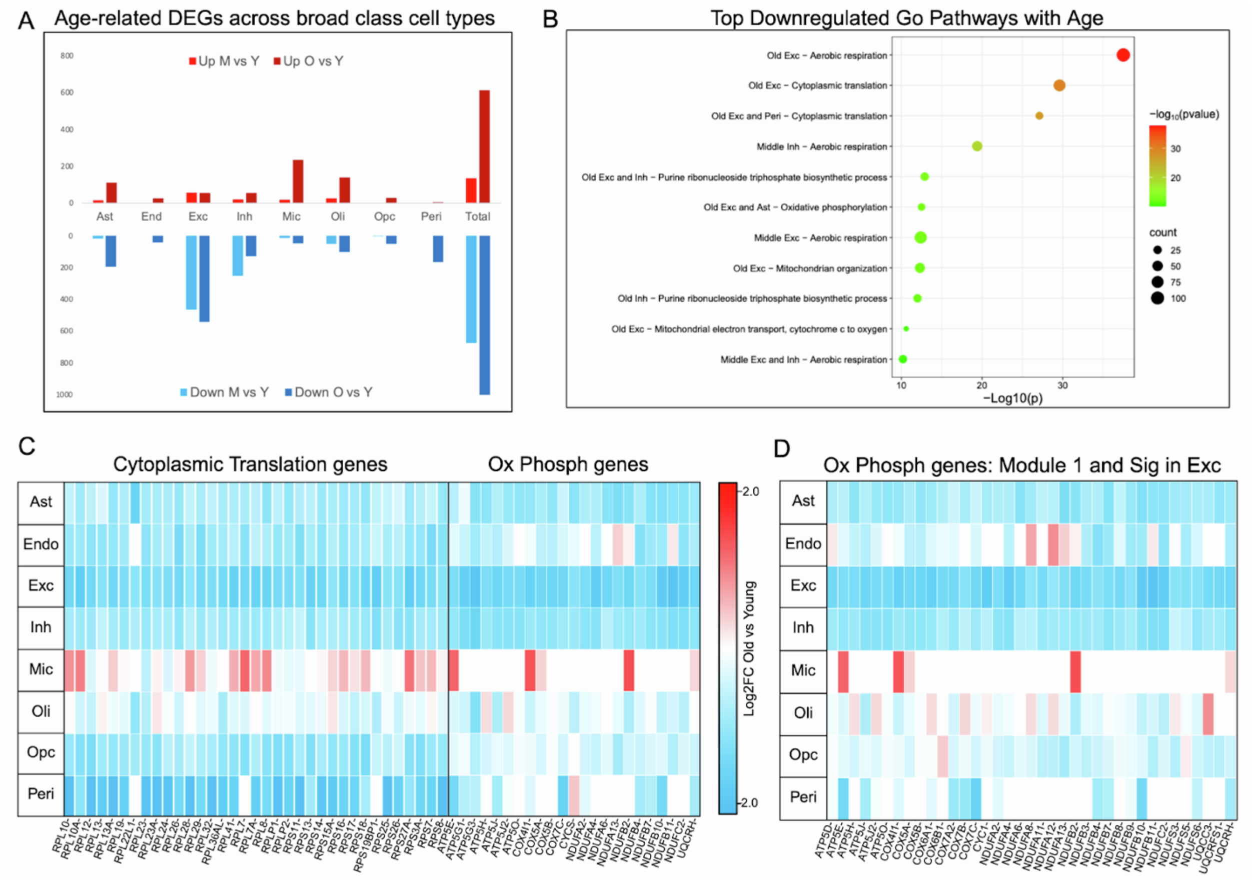

Figure S5 Summary of snRNA-seq DEG results, related to Figures 4 and 5

(A) Number of age-related DEGs for each broad class cell type. Red (blue) bars indicate the number of genes increasing (decreasing) with age, with different shades representing young vs middle and young vs old conditions (see legend).

(B) Top gene ontology biological process terms across significantly decreasing genes (adj. p-val<0.05, Log2FC<-1) from individual cell types (e.g. exc) and shared across pairs of cell types (e.g. exc and inh).

(C) Heatmap displaying genes significantly decreasing (adj. p-val<0.05, Log2FC<-1) across two or more broad class cell types comparing young and old groups, related to cytoplasmic translation (RPL/RPS) and oxidative phosphorylation. BH-adjusted p values were derived from linear modeling with *limma-voom*.

(D) Heatmap displaying genes significantly decreasing (adj. p-val<0.05, Log2FC<-1) in excitatory neurons comparing young and old groups that were both related to oxidative phosphorylation and present in spatial module M1. BH-adjusted p values were derived from linear modeling with *limma-voom*.

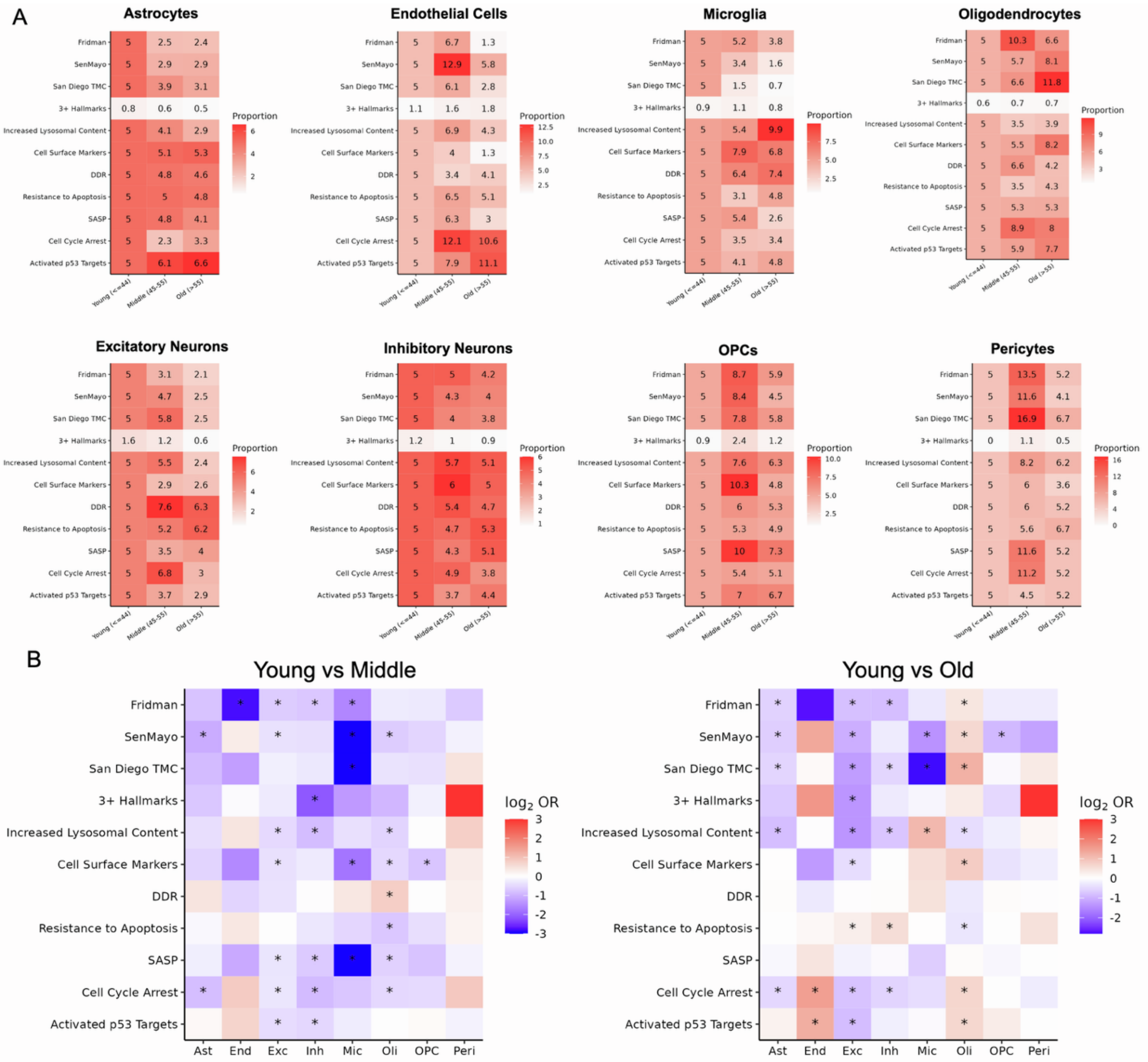

C

Cell type	CDKN1A_only	CDKN2A_only	both_expressed
Ast	0.7487	0.8041	0.0000
End	1.4394	0.5303	0.0000
Exc	0.8337	0.6804	0.0043
Inh	0.3970	1.9102	0.0058
Mic	3.6966	4.2727	0.2640
OPC	0.2948	3.4784	0.0236
Oli	0.4720	3.6298	0.0429
Peri	0.5563	0.1391	0.0000

Figure S6 Senescence hallmark scores by age and broad class, related to Figure 4
(**A**) Quantification of the raw proportion of cells for each broad class positive for each senescence hallmark. The young proportion is always at 5% (except for the "3+ hallmarks" measurements), as this is where the threshold is set for each individual broad class and hallmark.
(**B**) Heatmap displaying enrichment of senescent-positive nuclei between different labeled conditions across senescence hallmarks. Cells represent test statistics from testing for difference of proportions via t-test. Red (blue) cells indicate an increased (decreased) proportion of senescent nuclei in the old/middle (young) cohort. Reported p-values were derived from one-way ANOVA tests on MASC models[55]. Nominal p-values are annotated onto cells. *=p<1e-2.
(**C**) Tabulation of CDKN1A+, CDKN2A+, and double positive nuclei by percentage of each broad class cell type in our snRNA-seq dataset.

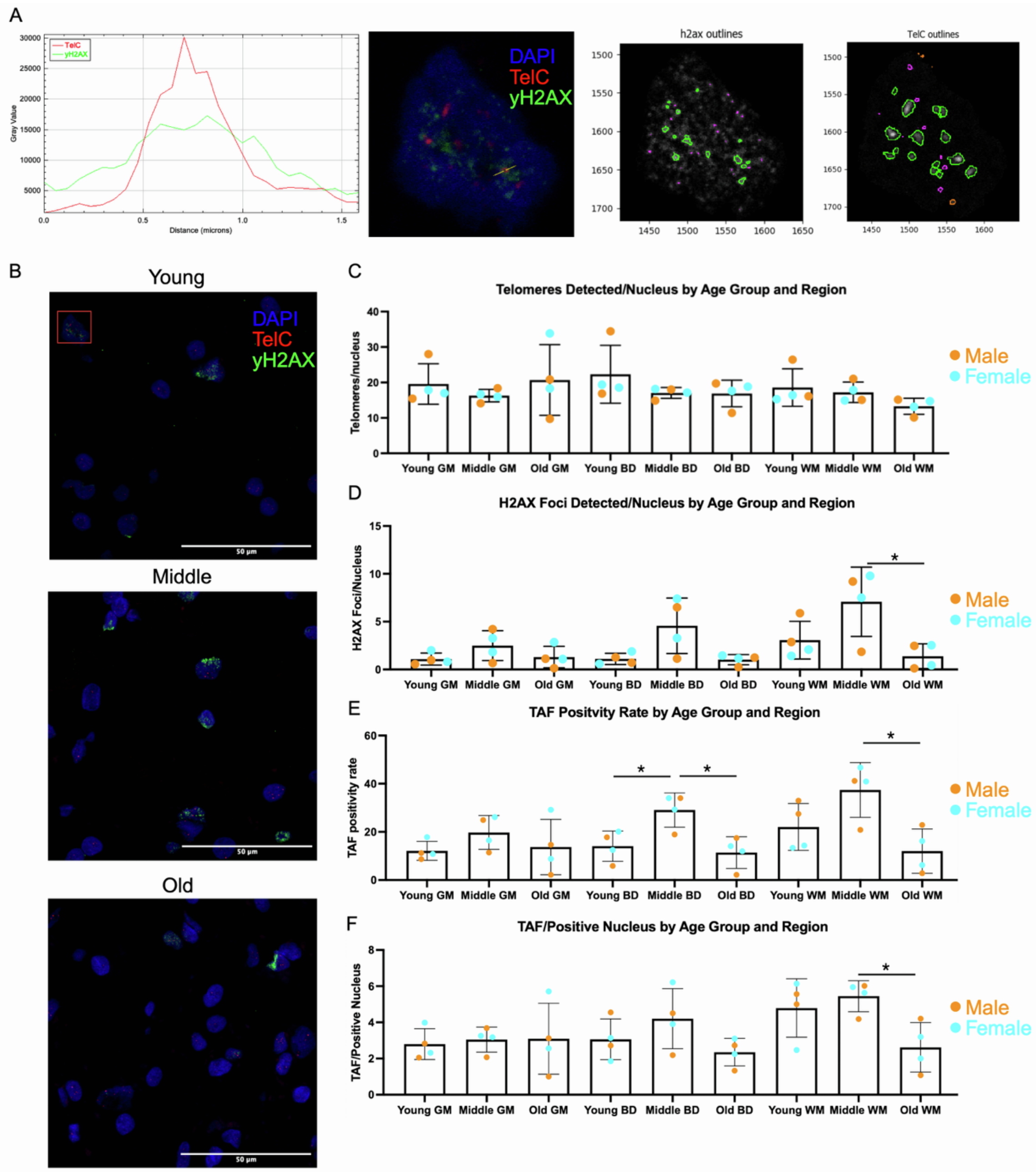

Figure S7 Telomere associated DNA damage response peaks in middle age group, dwindles later in aging, related to Figure 4

(A) dlPFC sections were stained for DAPI (blue), TelC (red), and yH2AX (green). Signal intensity of TelC and yH2AX along the yellow line in the middle left representative image are displayed in the far-left plot. Inflection of both signals represents an overlap in telomere and yH2AX foci, signifying TAF. Middle right and far right plots display CellProfiler calls for yH2AX and TelC foci, respectively (green outlines) within representative TAF positive cell (middle left).

(B) Representative images of TAF stain in young (n=4), middle (n=4), and old (n=4) subject tissues

(**C-F)** Quantification of **(C)** telomeres detected per nucleus, **(D)** yH2AX foci per nucleus, **(E)** TAF positivity percentage, and **(F)** TAF foci per TAF positive nucleus in the grey matter (GM), border of grey and white matter (BD), and white matter (WM) of the cortex across young (n=4), middle (n=4), and old (n=4) subjects. Error bars represent standard deviation, and dots represent individual measurements for each subject, colored by sex (see legend). Significance determined using unpaired 2-sided t test with Welch's correction. * = p-val<0.05.

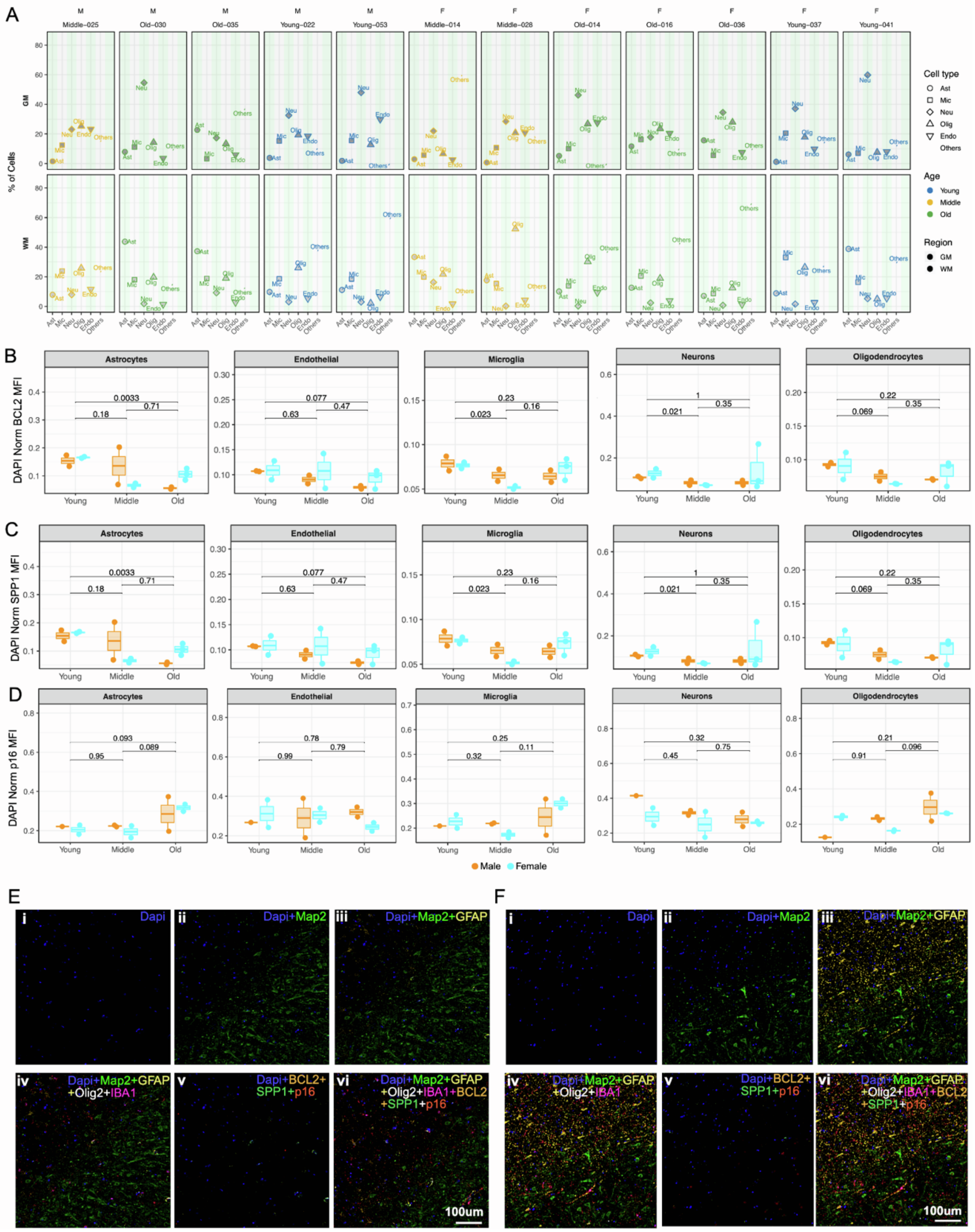

Figure S8 COMET extended results, related to Figure 4
(A) Proportion of cell types (GFAP+ astrocytes, CD31+ endothelial cells, IBA1+ microglia, MAP2+ neurons, and OLIG2+ oligodendrocytes) for each donor.
(B-D) Quantification of DAPI normalized MFI per cell of **(B)** BCL2, **(C)** SPP1, and **(D)** p16 in all cell types across young (n=4), middle (n=4) and old (n=5) donors. Significance for **(B-D)** derived using unpaired 2-sided t test with Welch's correction. Dots represent individual measurements for each subject colored by sex (see legend).
(E-F) Images of each cell type and sen-related protein target on representative young **(E)** and old **(F)** stained tissues.

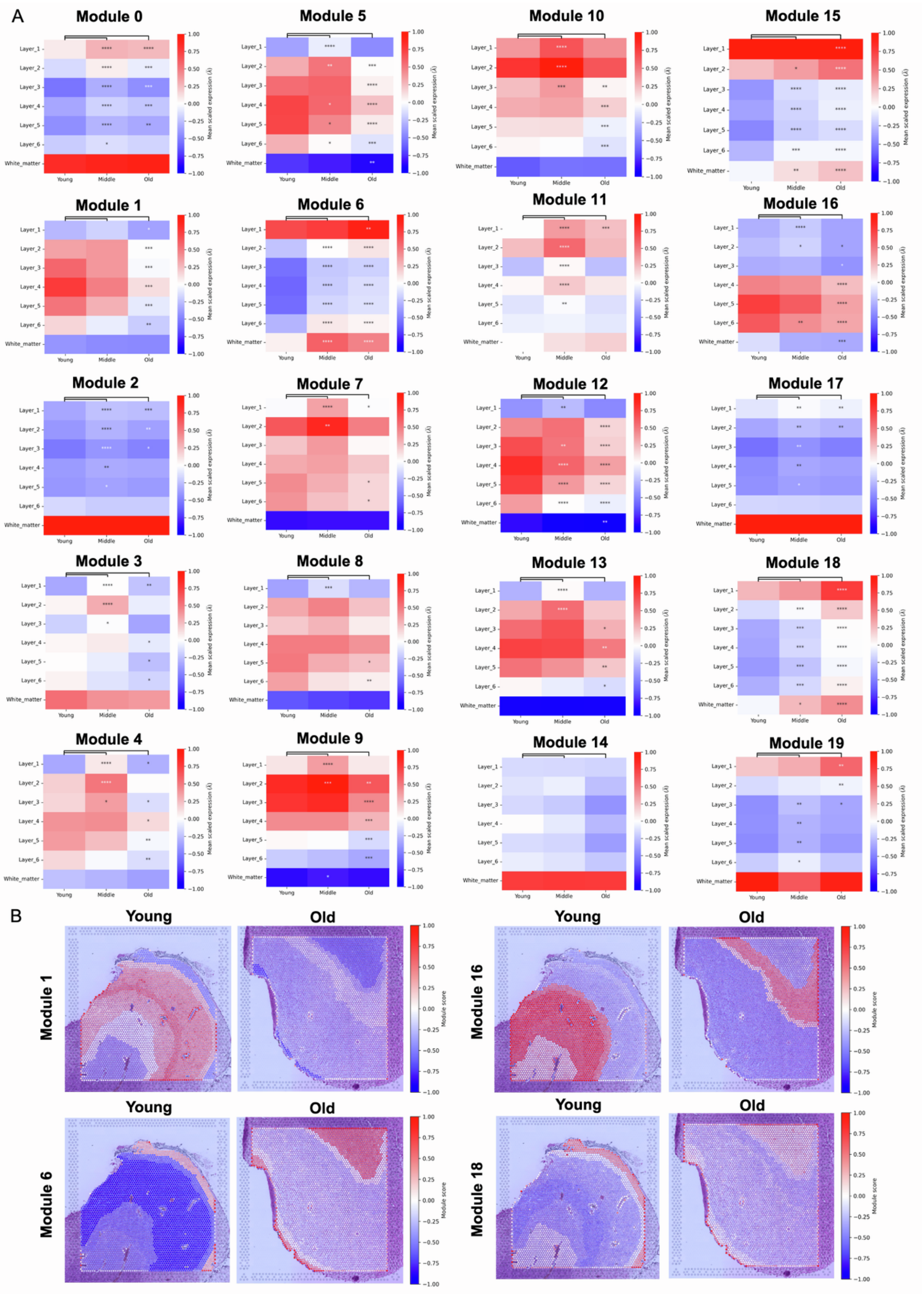

A
Module 0
Module 5
Module 10
Module 15
Module 1
Module 6
Module 11
Module 16
Module 2
Module 7
Module 12
Module 17
Module 3
Module 8
Module 13
Module 18
Module 4
Module 9
Module 14
Module 19
Layer_1
Layer_2
Layer_3
Layer_4
Layer_5
Layer_6
White_matter
Young
Middle
Old
Mean scaled expression (λ)
B
Young
Old
Module 1
Module 16
Module 6
Module 18
Module score

Figure S9 Pairwise spatial gene coexpression modules, related to Figure 5

(A) Average expression levels of each spatial module generated in Figure 5 (minimum 100 genes) by cortical layer and age groups, with differential testing results between old and middle versus young. For all modules, significant changes in scores across age groups for each layer were determined using Welch's t-test with BH FDR correction. Asterisks indicate level of significance: ****=p<1e-4***=p<1e-3 **=p<1e-2, *=p<.05.

(B) Representative spatial arrays from a young (left) and old (right) subject tissue for each module of interest displayed in Figure 5. Each ST spot is scored for module expression using the same scale as in A.

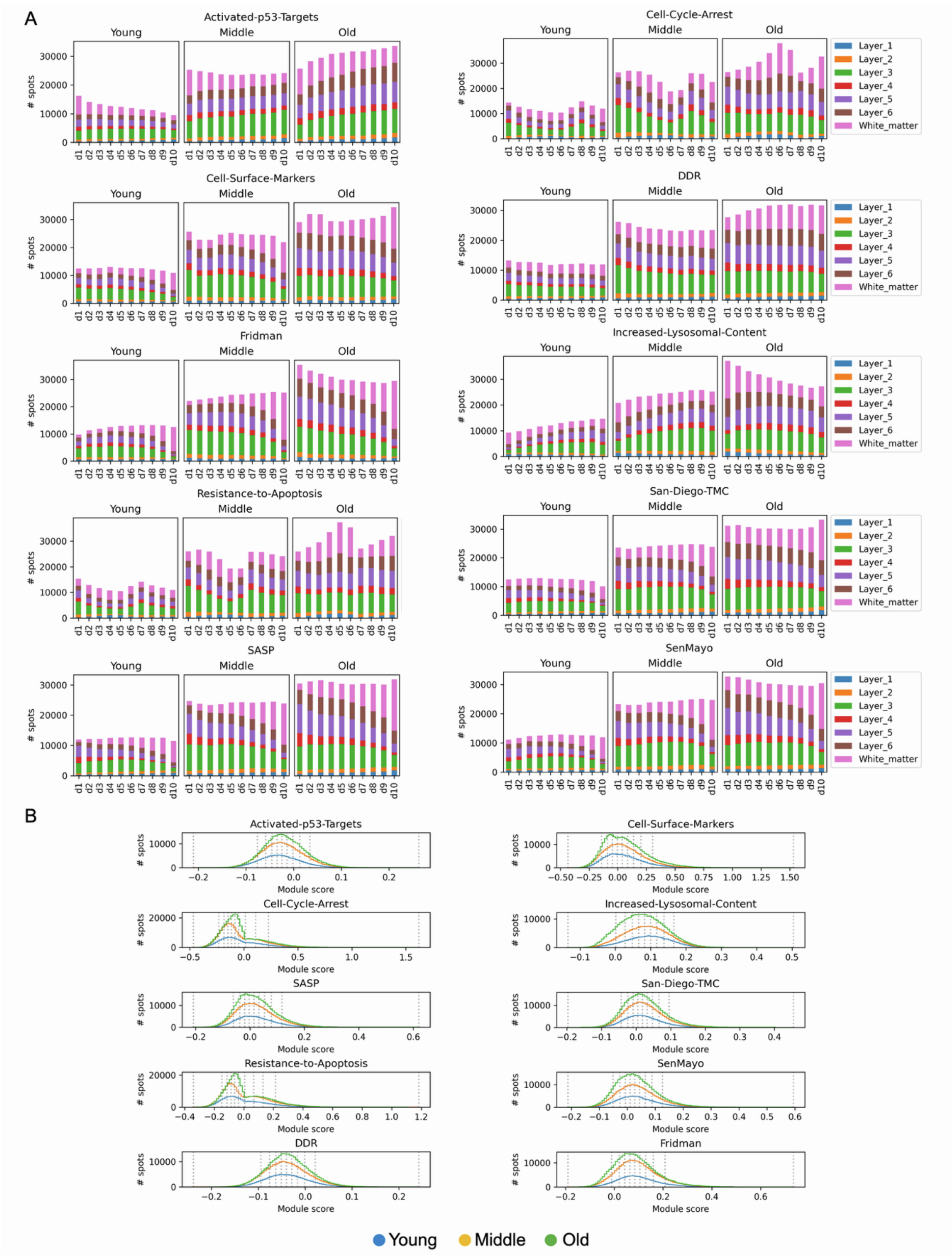

Figure S10 Extended senescence module local distribution analysis, related to Figure 6

(A) Distribution of spots scored at each decile for senescence hallmarks across young, middle, and old age groups, with stacked bar plots showing AAR locations of all spots separated by age and module expression. **(B)** Distribution of module scores (x-axes) across ST spots (y-axes) for senescence gene lists. ST spots are scored according to population-wide deciles (dotted vertical lines) and are broken down by age group (color code).

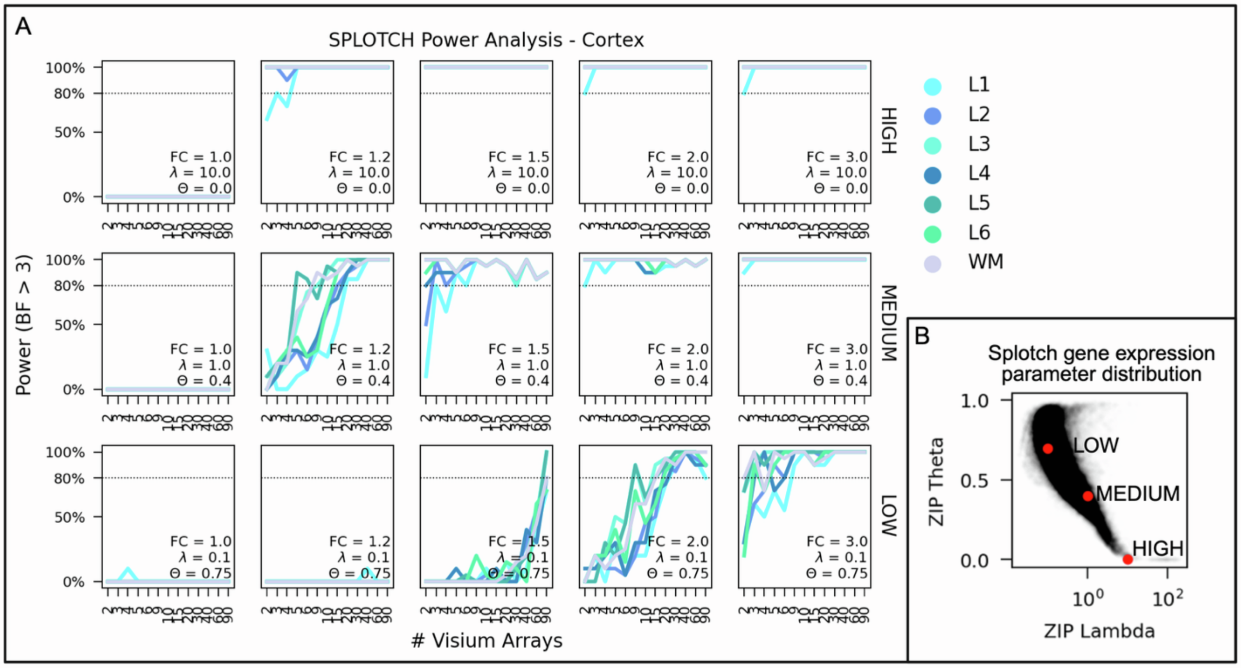

Figure S11 Power analysis for Visium ST results quantified by Splotch, related to STAR Methods
(A) Power curves displaying level of power (dotted line at 80% power) to detect changes in gene expression at threshold BF>3 for each array added per comparison group (i.e. young, middle, old) along the x axis of each plot. Each colored line represents the different annotated cortical layers that Splotch measures differential expression in (L1-WM). In addition to the number of arrays, we also tested power with varying levels of fold change (FC) in each plot from left to right, and varying gene expression parameters lambda ($\boldsymbol{\lambda}$) and theta ($\boldsymbol{\theta}$) in each plot from top to bottom.
(B) Plot displaying the maximum likelihood estimate of gene expression parameters ($\boldsymbol{\lambda}$) and ($\boldsymbol{\theta}$) for each transcript (black dots) and the parameters used for simulated LOW (~20th percentile), MEDIUM (~90th percentile) and HIGH (~99.9th percentile) expression genes (labeled red dots).
